# Global, Regional, and National Burden of Cardiovascular Disease, 1990-2021: Results From the 2021 Global Burden of Disease Study

**DOI:** 10.7759/cureus.74333

**Published:** 2024-11-24

**Authors:** Yue Wang, Xin Wang, Changfen Wang, Jianzhong Zhou

**Affiliations:** 1 Department of Cardiology, The First Affiliated Hospital of Chongqing Medical University, Chongqing, CHN; 2 Department of Cardiology, Qian Xi Nan People's Hospital, Zunyi, CHN

**Keywords:** cardiovascular disease, death, epidemiology, global burden, incidence, public health

## Abstract

Background

Cardiovascular diseases (CVD), including coronary artery disease, ischemic heart disease, stroke, cardiomyopathy, and atrial fibrillation and flutter, are the leading cause of mortality worldwide, resulting in significant economic and health costs. Recognizing trends and geographical differences in the global burden of CVD facilitates health authorities in particular nations to assess the disease burden and forecast future epidemiological trends. Public health authorities in each country can better understand the differences in disease data and, by learning from the experiences and practices of successful countries and considering the characteristics of their diseases, allocate health resources more rationally and formulate more targeted healthcare strategies to reduce the disease burden. This study aims to comprehensively assess CVD trends and geographic variations from 1990 to 2021.

Methods

This study focuses on analyzing global trends in the epidemiology of all-age CVD incidence and death over the past 30 years. A vital registration system, cause-of-death inference records, and a cause-of-death ensemble model (CODEm) were used to estimate cause-specific mortality for CVD, with CODEm estimates adjusted using a cause-of-death correction (CoDCorrect) algorithm. Incidence data were extracted from insurance claims and inpatient discharge sources and analyzed with Disease Modeling Meta-Regression, Version 2.1 (DisMod-MR 2.1). Data were extracted from the 2021 Global Burden of Disease Study (GBD 2021) on the number of incident cases and deaths, as well as age-standardized incidence rates (ASIR) and age-standardized death rates (ASDR) for CVD for each year from 1990 to 2021. We visualized and reported this data at the global, regional, and national levels. To explore the association between the burden of CVD and sociodemographic factors, we used the sociodemographic index (SDI), which categorizes the world's 204 nations into five SDI regions. Because the GBD results are a combination of data and estimates, 95% uncertainty intervals (UI) are provided for each count and rate (per 100,000 populations).

Results

Globally, the number of CVD incident cases and deaths increased from 34.74 million and 12.33 million in 1990 to 66.81 million and 19.42 million in 2021, representing a 92.3% and 57.5% rise, respectively. However, the global ASIR and ASDR for CVD have decreased by 10.4% and 34.3%, respectively, since 1990. Notably, among the 21 regions of the world, the ASIR for CVD is on a downward trend from 1990 to 2021, except for East and Central Asia, where the ASIR for CVD increased by 3% and 14.3%, respectively. Similarly, the global ASDR for CVD is only on an upward trend in sub-Saharan Africa, increasing by 12%, while all other regions are on a downward trend. Among the five SDI regions, the high SDI region has much lower ASIR and ASDR compared to the world average, and these rates have decreased significantly over the years.

Conclusion

Despite a significant increase in the number of CVD incident cases and deaths worldwide over the last three decades, ASIR and ASDR have been declining. Over the past 30 years, both ASIR and ASDR for CVD have declined significantly in high SDI areas, while CVD continues to pose a serious public health threat in regions with low SDI.

## Introduction

Cardiovascular diseases (CVD), primarily ischemic heart disease and stroke, are the leading cause of global mortality and a major contributor to disability [1]. As of 2017, more than 17 million people have died from CVD, and over 360 million disability-adjusted life years (DALYs) have been attributed to it, making CVD the highest-burden disease in the world [2]. The distribution of the CVD burden is influenced by a variety of factors, including socioeconomic status, lifestyles, the prevalence of risk factors, and access to healthcare [3]. The primary risk factors for CVD vary by region; for example, CVD mortality from smoking is highest in Oceania, CVD deaths from high-sodium diets are most common in Asia, and CVD deaths from high systolic blood pressure are highest in Central Asia. In contrast, particulate matter pollution and low-fruit, low-vegetable diets pose the most danger in low- and middle-income countries [2]. The prevalence of CVD increased rapidly in the first half of the 20th century as a result of industrialization, urbanization, rising prosperity, and social unrest in high-income countries, followed by a significant decrease in CVD mortality in the second half of the century. As globalization and population aging continue, changes in lifestyle and dietary trends will have a significant influence on the burden of CVD. Previous studies published under the Global Burden of Disease Study (GBD) 2019 have highlighted geographic differences in the burden of CVD [4]. GBD 2021 is an upgrade and expansion of GBD 2019, using advanced statistical methods and models to improve data quality and dependability; the time range has been expanded from 1990 to 2021, providing a longer period to analyze health trends. However, since the GBD 2021 database was updated, there has been a lack of analysis of the most recent data on CVD incidence and deaths, as well as comparisons between the five sociodemographic index (SDI) regions. In this study, we retrieved data on CVD incidence and mortality from the GBD 2021 and conducted a thorough evaluation and comparison at the global, regional, and national levels. Our objective was to examine worldwide trends and regional variations in CVD incidence and death rates from 1990 to 2021. This analysis will help public health authorities and stakeholders identify data gaps, assess unmet needs, and effectively implement and scale up intervention services.

## Materials and methods

Data source

All data used in this study can be extracted through the Global Health Data Exchange query tool (https://ghdx.healthdata.org/gbd-2021). The Institute for Health Metrics and Evaluation (IHME) at the University of Washington leads and updates the database. Population censuses, household surveys, civil registration and vital statistics, disease registries, healthcare utilization, air pollution monitoring, satellite imaging, disease notification, and other sources provided input data [4]. Meanwhile, because the GBD database incorporates a variety of raw data sources, the quality of the individual studies that contribute to the data may have an impact on the accuracy and dependability of the estimates. Details of the GBD study's data sources, modeling techniques, and tools have already been published previously [5-7].

Case definition

According to the International Classification of Diseases (ICD) system, CVD is defined in 11 major etiologic categories: ischemic heart disease, stroke, hypertensive heart disease, peripheral arterial disease, atrial fibrillation and atrial flutter, cardiomyopathy and myocarditis, endocarditis, rheumatic heart disease, non-rheumatic valvular heart disease, aortic aneurysm, and other cardiovascular and circulatory diseases [2,4,8]. Although ICD codes are commonly recognized for obtaining information on CVD cases, the version utilized changes by location and over time, resulting in the overestimation or underestimation of CVD.

SDI

The GBD study provides the SDI, which is an equally weighted geometric mean of per capita income, educational attainment, and total fertility. SDI scores range from 0 to 1, and based on SDI values, the world is divided into five SDI regions: low SDI (SDI < 0.25), medium-low SDI (0.25 ≤ SDI < 0.50), medium SDI (0.50 ≤ SDI < 0.75), medium-high SDI (0.75 ≤ SDI < 0.90), and high SDI (SDI ≥ 0.90). Generally, the higher the SDI value, the higher the per capita income, the higher the level of education, and the lower the fertility rate of women under 25 years of age. This also indirectly reflects the level of development of the region, the availability of medical resources, and the public's awareness of diseases. Based on the 2021 SDI values, the 204 countries and territories were categorized into five SDI regions: low SDI (e.g., Afghanistan, Niger, and Somali), medium-low SDI (e.g., Bangladesh, Cameroon, and Ethiopia), medium SDI (e.g. China, India, and Egypt), medium-high SDI (e.g., Chile, Malaysia, and Turkey), and high SDI (e.g. Australia, Japan, and Norway).

Statistical analysis

Our study aims to analyze global trends and regional differences in CVD incidence and death from 1990 to 2021. A vital registration system, cause-of-death inference records, and a cause-of-death ensemble model (CODEm) were used to estimate cause-specific mortality for CVD, with CODEm estimates adjusted using a cause-of-death correction (CoDCorrect) algorithm [9]. Incidence data were extracted from insurance claims and inpatient discharge sources and analyzed with Disease Modeling Meta-Regression, Version 2.1 (DisMod-MR 2.1) [9]. At each modeling phase, 500-1,000 draws were made from the posterior distribution to establish the 95% uncertainty ranges for the final load estimates. To ensure standardized comparisons between populations with different age structures and over time, we introduced age-standardized rates (ASR), which primarily include age-standardized incidence rates (ASIR) and age-standardized death rates (ASDR). We extracted data from the GBD 2021 on CVD incidence and death from 1990 to 2021 at the global, 21 regional, and 204 country levels, all of which were collated and visualized using R (Version 4.3.3, RStudio, R Foundation for Statistical Computing, Vienna, Austria (https://www.R-project.org/)).

## Results

Global trends of CVD

It can be observed from Table 1 that, globally, the number of CVD incident cases increased from 34.74 million in 1990 to 66.81 million in 2021, representing a 92.31% increase. During the same period, the ASIR of CVD fell from 878.8 cases per 100,000 in 1990 to 787 cases per 100,000 in 2021, a decline of 10.4%. Additionally, the number of CVD deaths rose from 12.33 million in 1990 to 19.42 million in 2021, an increase of 57.5%. Over the same period, the ASDR of CVD decreased from 358.1 cases per 100,000 in 1990 to 235.2 cases per 100,000 in 2021, marking a 34.3% reduction (Table 1 provides detailed data at the global level).

**Table 1:** ASIR, ASDR, and the number of incident cases and deaths of cardiovascular disease in 1990 and 2021 at the global level and for 21 regions ASIR: age-standardized incidence rates; ASDR: age-standardized death rates; ASR: age-standardized rates; 95% UI: 95% uncertainty intervals Number of incident cases=numbers×100,000 Number of deaths=numbers×100,000 Percentage change in ASR from 1990 to 2021=(the ASR in 2021-the ASR in 1990)/the ASR in 1990

	Incidence					Deaths				
	Numbers in 100,000 (95% UI)		ASR per 100,000 (95% UI)		Percentage change in ASR from 1990 to 2021	Numbers in 100,000 (95% UI)		ASR per 100,000 (95% UI)		Percentage change in ASR from 1990 to 2021
	1990	2021	1990	2021		1990	2021	1990	2021	
Global	347.4 (318.5, 379.8)	668.1 (609.1, 731)	878.8 (808.2, 959.9)	787 (719.7, 859.7)	-10.4 (-11.5, -9.4)	123.3 (116.2, 127.9)	194.1 (177.8, 206.7)	358.1 (333.7, 372.6)	235.2 (214.6, 250.5)	-34.3 (-37.8, -30.7)
Australasia	1.8 (1.7, 1.9)	2.9 (2.6, 3.2)	772.1 (719.9, 830.2)	553 (505.7, 607.8)	-28.4 (-31.3, -25.1)	0.6 (5.8, 6.5)	0.6 (0.5, 0.6)	280 (256.8, 291.9)	94.2 (80.8, 101.2)	-66.4 (-68.6, -65)
Caribbean	2.1 (2, 2.4)	3.9 (3.5, 4.3)	796.1 (728.5, 874.5)	736.4 (671.4, 810.1)	-7.5 (-8.9, -6)	0.8 (7.8, 8.5)	1.2 (1.1, 1.4)	342 (324.2, 354.2)	228.4 (203.3, 254.9)	-33.2 (-40.2, -25.8)
Central Asia	5.2 (4.8, 5.7)	9.7 (9, 10.5)	1120.4 (1033.2, 1214.4)	1280.2 (1202.2, 1376.2)	14.3 (11.6, 17)	2.1 (20.4, 22.0)	2.9 (2.7, 3.2)	512.9 (484.2, 529.8)	436.1 (395.7, 474.5)	-15 (-21.7, -8)
Central Europe	13.4 (12.3, 14.5)	15.1 (14.1, 16.3)	935.3 (867.4, 1006.7)	700 (653.4, 751.2)	-25.2 (-27.2, -23.3)	7.2 (69.6, 73.8)	6.8 (6.2, 7.3)	542.8 (516.1, 555.8)	288 (262.8, 306.1)	-46.9 (-49.9, -44.1)
Central Latin America	6.2 (5.7, 6.8)	15.5 (14, 17.3)	694.6 (630.3, 771.4)	620.1 (558.5, 689.3)	-10.7 (-12, -9.6)	1.7 (15.8, 16.9)	3.9 (3.5, 4.3)	230.6 (217.5, 236.6)	163.7 (145.6, 180.7)	-29 (-35.3, -22.3)
Central Sub-Saharan Africa	2.4 (2.2, 2.7)	5.7 (5.1, 6.3)	853.2 (775.4, 937.9)	786.4 (721.7, 859.2)	-7.8 (-10.5, -5.5)	0.7 (6.2, 8.5)	1.5 (1.2, 1.8)	410.9 (351.6, 468.3)	361.4 (292.1, 444.2)	-12.1 (-27.7, 7.2)
East Asia	65 (59.6, 71.3)	165.8 (149.4, 183.7)	785.6 (719.7, 858.4)	809.3 (734.4, 888.7)	3 (0.8, 5.2)	25.8 (228.1, 286.0)	52.3 (44.5, 60.6)	403.3 (358.4, 445.9)	276.2 (235.1, 318.3)	-31.5 (-42.4, -18.1)
Eastern Europe	30.8 (27.3, 34.6)	39.1 (34.2, 44.5)	1177.9 (1049, 1316.1)	1135.6 (1000.2, 1279.3)	-3.6 (-5.9, -1.1)	14.0 (133.6, 142.3)	14.9 (13.5, 16.2)	570.1 (540.1, 583.5)	421.6 (381.3, 458.3)	-26 (-31.4, -20.5)
Eastern Sub-Saharan Africa	7.6 (6.8, 8.4)	16.8 (15, 18.7)	782.8 (708.8, 860.2)	737.7 (670, 813.8)	-5.8 (-7.2, -4.5)	2.1 (19.5, 22.9)	3.6 (3.2, 4.0)	330.3 (302.7, 356.9)	256 (231.4, 285.4)	-22.5 (-29.8, -13.4)
High-income Asia Pacific	11.4 (10.7, 12.2)	18.2 (16.8, 19.6)	576.2 (541.2, 614.8)	422.7 (395.2, 451.9)	-26.6 (-28.6, -24.8)	3.6 (32.6, 37.5)	4.6 (3.6, 5.1)	204.1 (182.1, 214.9)	73.4 (61.3, 79.8)	-64 (-66.4, -62.5)
High-income North America	34.4 (30.5, 38.2)	42.9 (40.5, 45.6)	986.6 (881.2, 1101.4)	670.2 (635.1, 708.8)	-32.1 (-37, -27.2)	9.4 (85.0, 98.8)	9.8 (8.5, 10.5)	260.4 (234.8, 272.6)	139.5 (122.5, 148.1)	-46.4 (-48.2, -45.3)
North Africa and the Middle East	22.9 (20.6, 25.8)	56.6 (51, 63.8)	1336.5 (1212.7, 1504.3)	1232.3 (1117.9, 1378.3)	-7.8 (-9.7, -5.8)	7.5 (69.9, 79.6)	13.5 (12.1, 15.0)	529.2 (486.4, 560.6)	363.5 (322.3, 397.3)	-31.3 (-37.1, -25)
Oceania	0.3 (0.2, 0.3)	0.6 (0.6, 0.7)	821.1 (735.9, 920.8)	807.5 (724.6, 906.9)	-1.7 (-3.5, 0.2)	0.1 (0.9, 1.3)	0.2 (0.2, 0.3)	449.5 (384.7, 525.9)	377.8 (321.2, 438.3)	-15.9 (-29.1, -0.2)
South Asia	57.4 (50.5, 65.1)	135.7 (119.7, 153.6)	957 (838.1, 1087.4)	912.2 (805.6, 1032.6)	-4.7 (-5.8, -3.6)	15.6 (140.3, 167.4)	36.7 (33.8, 39.6)	302.5 (271.1, 326.8)	278.1 (256.5, 299.6)	-8 (-17.7, 2.6)
Southeast Asia	18.3 (17, 19.7)	43.1 (40, 46.7)	699.3 (649.8, 758.4)	675.1 (627.4, 730.3)	-3.5 (-4.7, -2.2)	7.8 (70.8, 84.8)	17.2 (15.6, 18.6)	355.5 (318.9, 388.7)	302.9 (273.2, 326.1)	-14.8 (-23.9, -3.4)
Southern Latin America	3.5 (3.3, 3.8)	4.8 (4.5, 5.2)	770 (721.9, 826.2)	575.2 (540.1, 617.2)	-25.3 (-27.3, -23.2)	1.3 (12.7, 13.7)	1.2 (1.1, 1.3)	315.6 (297.1, 324.9)	137.6 (126.1, 144.6)	-56.4 (-58, -54.9)
Southern Sub-Saharan Africa	2.7 (2.4, 3)	4.9 (4.4, 5.5)	864.6 (773.9, 965.8)	815.5 (732.5, 910.4)	-5.7 (-7.1, -4.2)	0.6 (5.3, 6.4)	1.3 (1.2, 1.4)	245.7 (217.5, 269.4)	275.1 (256.6, 292.1)	12 (4.1, 25.2)
Tropical Latin America	7 (6.5, 7.6)	13.6 (12.5, 14.7)	671.3 (624.6, 724.5)	547.1 (505, 592.5)	-18.5 (-20.3, -16.6)	2.6 (25.0, 27.0)	3.9 (3.5, 4.1)	331.7 (308, 343)	154.6 (140.5, 162.8)	-53.4 (-55.3, -52.1)
Western Europe	45.6 (42.8, 48.7)	51 (48, 54.6)	800.6 (757.2, 854.4)	576.7 (542, 619.3)	-28 (-29.6, -26.4)	16.7 (152.4, 173.1)	12.6 (10.6, 13.7)	280.7 (256.2, 292.2)	106.8 (91.8, 114.4)	-62 (-64.3, -60.7)
Western Sub-Saharan Africa	8.1 (7.3, 8.9)	18.8 (17, 20.7)	785.2 (705, 874.6)	765.8 (689, 848.5)	-2.5 (-4.1, -1.1)	2.7 (23.9, 29.7)	4.7 (4.0, 5.3)	354.3 (315.9, 393.3)	291.9 (260.9, 326.2)	-17.6 (-27.7, -5.5)
Latin America and the Caribbean	16.8 (15.5, 18.2)	36.3 (33.2, 39.9)	686.8 (632.6, 750.4)	590.9 (542, 647.6)	-14 (-15.2, -12.7)	5.5 (52.2, 56.1)	9.6 (8.7, 10.4)	281.7 (264.6, 289.5)	160.9 (145.5, 172.7)	-42.9 (-46.1, -39.4)

The number of incidences and deaths from CVD continues to increase globally as a result of a combination of the global population explosion of the past 30 years, the progression of population aging, and advances in diagnostic technology. At the same time, developments in medical technology, the adoption of public health measures, effective risk factor control, and the improvement of socioeconomic situations have all contributed to a decrease in the ASIR and ASDR of CVD.

Regional and national trends of CVD

In 2021, the regions with the highest number of CVD incident cases were East Asia (16.6 million cases) and South Asia (13.6 million cases), which accounted for 24.9% and 20.4%, respectively, of the total number of global CVD incident cases. In East Asia, China has the most incident cases, with 16 million instances, accounting for 24% of the global total number of CVD incident cases. In South Asia, India has the most incident cases, with 10.8 million instances, accounting for 16.2% of the total number of CVD incident cases worldwide (Table 1). During the same duration, the areas with the largest number of CVD deaths were East Asia (5.23 million cases) and South Asia (3.67 million cases), accounting for 26.8% and 19.1%, respectively, of total CVD deaths. China had the most deaths in East Asia, accounting for 5.1 million cases and 26.2% of overall CVD deaths globally. India had the largest number of deaths in South Asia, with 2.9 million cases, accounting for 14.9% of global CVD deaths (Table 1).

**Figure 1:**
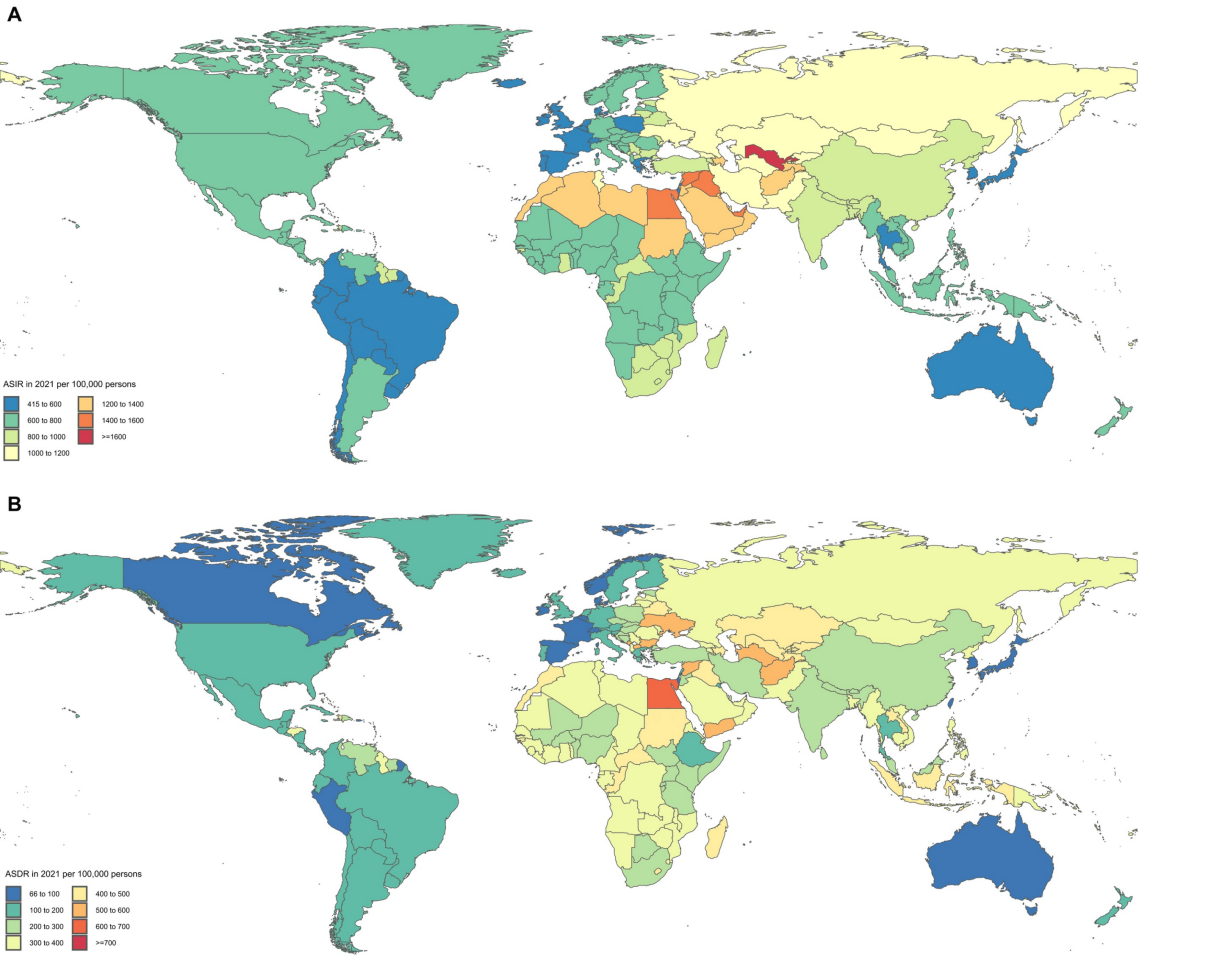
Global trends in ASIR (A) and ASDR (B) of cardiovascular disease in 204 countries and territories in 2021 ASIR: age-standardized incidence rates; ASDR: age-standardized death rates

**Figure 2:**
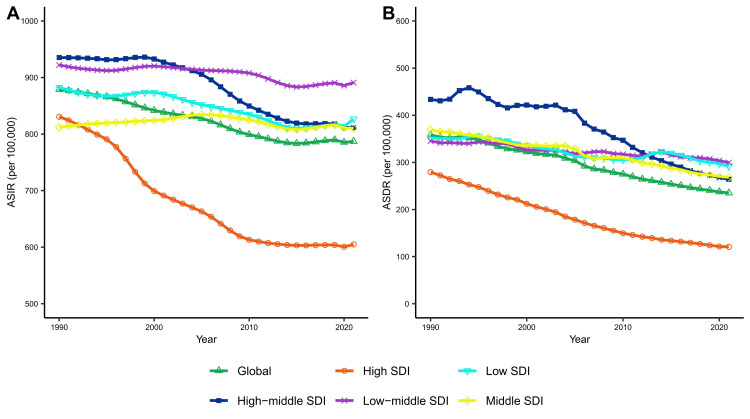
Global and five SDI region trends in ASIR (per 100,000 population) (A) and ASDR (per 100,000 population) (B) of cardiovascular disease from 1990 to 2021 (each point indicates the age-standardized rate for that year) ASIR: age-standardized incidence rates; ASDR: age-standardized death rates; SDI: sociodemographic Index

In 2021, the highest ASIR for CVD are in Central Asia (1280.2 per 100,000), North Africa and the Middle East (1232.3 per 100,000), and Eastern Europe (1135.6 per 100,000), and the lowest are in high-income Asia Pacific (422.7 per 100,000), Tropical Latin America (547.1 per 100,000), and Australasia (553 per 100,000) (Table 1). The countries with the highest ASIR for CVD were Uzbekistan (1721.9 per 100,000), the Syrian Arab Republic (1480.4 per 100,000), and the United Arab Emirates (1464.5 per 100,000), and the lowest were Portugal Republic (415.7 per 100,000), Korea (423.5 per 100,000), and Japan (424.2 per 100,000) (Table 2 and Figure 1). During the same period, the highest ASDR for CVD was found in Central Asia (436.1 per 100,000), Eastern Europe (421.6 per 100,000), and Oceania (377.8 per 100,000), while the lowest was found in high-income Asia Pacific (73.4 per 100,000), Australasia (94.2 per 100,000), and Western Europe (106.8 per 100,000) (Table 1). The countries with the highest ASDR for CVD were Nauru (748.3 per 100,000), Egypt (612.1 per 100,000), and Afghanistan (567.4 per 100,000), and the lowest were the Republic of San Marino (66.9 per 100,000), Japan (72.5 per 100,000), and Singapore (75.8 per 100,000) (Table 3 and Figure 2).

**Table 2:** The number of incident cases of cardiovascular disease and their ASIR for 204 countries worldwide in 1990 and 2021 and the percentage change in ASIR from 1990 to 2021 were calculated ASIR: age-standardized incidence rates (per 100,000); 95% UI: 95% uncertainty intervals The number of incident cases=incident cases×1 Percentage change in the ASIR per 100,000=(the ASIR in 2021-the ASIR in 1990)/the ASIR in 1990

	Incident cases (95% UI)		ASIR (95% UI) (per 100,000)		Percentage change in the ASIR per 100,000
	1990	2021	1990	2021	
Global	34742351 (31845969, 37984786)	66809837 (60907604, 73102151)	878.8 (808.2, 959.9)	787 (719.7, 859.7)	-10.4 (-11.5, -9.4)
Andean Latin America	141785 (128822, 155290)	331735 (301294, 366360)	587.6 (538.8, 648.2)	545.8 (495.3, 604.3)	-7.1 (-8.9, -5.1)
Plurinational State of Bolivia	23674 (21431, 26133)	54876 (49654, 60634)	626.8 (569.6, 694.4)	581.2 (528.2, 641.2)	-7.3 (-9.8, -5)
Republic of Ecuador	37457 (33916, 41098)	95814 (87115, 105871)	597.3 (544.6, 660)	577.4 (525.3, 637.6)	-3.3 (-5.7, -0.8)
Republic of Peru	80654 (73144, 88298)	181045 (164618, 199669)	572.4 (523.8, 631.2)	523.9 (474.7, 580.1)	-8.5 (-11.2, -5.6)
Australasia	179287 (166588, 193524)	287698 (263280, 315014)	772.1 (719.9, 830.2)	553 (505.7, 607.8)	-28.4 (-31.3, -25.1)
Australia	144380 (135657, 154532)	237889 (217030, 261342)	747.2 (703.1, 799)	543.7 (497.1, 599.7)	-27.2 (-31.1, -22.6)
New Zealand	34907 (30727, 39748)	49809 (45922, 54467)	896.8 (794.5, 1020.3)	602.3 (556.1, 652.7)	-32.8 (-36.9, -28.2)
Caribbean	214934 (196274, 235252)	389258 (354166, 429816)	796.1 (728.5, 874.5)	736.4 (671.4, 810.1)	-7.5 (-8.9, -6)
Antigua and Barbuda	426 (379, 481)	758 (668, 870)	766.9 (680.3, 861.7)	748.7 (662.6, 847)	-2.4 (-5, 0.2)
Commonwealth of the Bahamas	1314 (1174, 1460)	3029 (2667, 3458)	769.5 (685.3, 868.6)	764.4 (675.2, 867)	-0.7 (-3.4, 2)
Barbados	2171 (1925, 2464)	3602 (3149, 4136)	760 (679.7, 852.9)	766 (680.1, 866.5)	0.8 (-1.9, 3.6)
Belize	791 (707, 880)	2323 (2069, 2634)	705.6 (623.4, 798.2)	722.4 (637.5, 824.1)	2.4 (-0.3, 5.3)
Bermuda	419 (369, 476)	852 (736, 994)	684.8 (605.7, 779.1)	653.7 (571.7, 749.6)	-4.5 (-7.3, -1.4)
Republic of Cuba	83816 (76435, 91976)	124061 (112720, 137800)	818.9 (751.2, 897.7)	689.6 (630.2, 759.2)	-15.8 (-18.3, -13)
Commonwealth of Dominica	460 (410, 520)	597 (521, 682)	751.2 (667.8, 847.2)	752.5 (662.1, 853.2)	0.2 (-2.7, 2.8)
Dominican Republic	30587 (27538, 33955)	77964 (71336, 85287)	712.4 (641.7, 788.2)	767.9 (701, 837.5)	7.8 (3.4, 12.5)
Grenada	626 (560, 701)	865 (767, 982)	821.6 (735, 917.3)	804 (717, 906)	-2.1 (-5.2, 0.6)
Republic of Guyana	4160 (3780, 4561)	5427 (4885, 6082)	950.8 (858.9, 1058.1)	843.7 (756.4, 943)	-11.3 (-13.6, -8.9)
Republic of Haiti	32305 (29433, 35430)	67163 (60777, 73994)	909.3 (829, 992.5)	840.1 (767.6, 918.6)	-7.6 (-10.4, -4.9)
Jamaica	14046 (12594, 15711)	23355 (20749, 26286)	737.6 (656.8, 829.8)	749.8 (665.9, 842.5)	1.7 (-1.1, 4.3)
Puerto Rico	24260 (21643, 27195)	42153 (37144, 47911)	682.4 (609.3, 761.7)	617.3 (544.8, 698.5)	-9.5 (-13.5, -6)
Saint Kitts and Nevis	302 (267, 343)	480 (420, 551)	831.5 (744.2, 934.6)	725.7 (638.1, 824.5)	-12.7 (-15.3, -9.7)
Saint Lucia	816 (731, 909)	1785 (1579, 2027)	872.9 (777.9, 978.4)	785.2 (699.6, 882.4)	-10.1 (-12.5, -7.7)
Saint Vincent and the Grenadines	604 (541, 678)	1016 (895, 1152)	779.7 (697.6, 879.7)	750.5 (668.9, 844.2)	-3.7 (-6.1, -1)
Republic of Suriname	2230 (2001, 2501)	4943 (4370, 5597)	812.3 (724.8, 916.5)	801.4 (712, 905.4)	-1.3 (-4, 1.5)
Republic of Trinidad and Tobago	7776 (6985, 8681)	14526 (12671, 16572)	886.3 (794.3, 994.8)	797.7 (704, 902.1)	-10 (-12.6, -7.3)
United States Virgin Islands	549 (486, 625)	1186 (1028, 1373)	660.4 (581.5, 755.4)	685.7 (602.3, 781.9)	3.8 (0.6, 6.6)
Central Asia	524031 (482897, 571795)	965082 (898449, 1048195)	1120.4 (1033.2, 1214.4)	1280.2 (1202.2, 1376.2)	14.3 (11.6, 17)
Republic of Armenia	28771 (25858, 32271)	40763 (36203, 46207)	1092.8 (986.2, 1212)	976 (869.4, 1092.1)	-10.7 (-14, -7.5)
Republic of Azerbaijan	54086 (49350, 59616)	115026 (106165, 125594)	1088.1 (994.3, 1193)	1267.9 (1179, 1367.6)	16.5 (10.6, 22.3)
Georgia	76781 (71271, 84336)	63829 (57791, 70504)	1301.7 (1215.7, 1416.7)	1097.4 (995.2, 1205.6)	-15.7 (-19.8, -10.7)
Republic of Kazakhstan	136008 (125659, 147550)	172143 (157680, 189509)	1120.9 (1040.7, 1215)	1032.1 (951.4, 1122.2)	-7.9 (-11.1, -4.7)
Kyrgyz Republic	32976 (29936, 36607)	47608 (42989, 52748)	1083 (979.5, 1210.2)	1005.4 (914.6, 1110.5)	-7.2 (-10.9, -3.6)
Mongolia	13968 (12620, 15613)	26603 (23827, 29685)	1259.2 (1125.7, 1416.3)	1185.2 (1060.3, 1322.5)	-5.9 (-9, -2.5)
Republic of Tajikistan	30587 (27728, 34201)	68040 (62426, 74963)	1033.4 (925.8, 1158.9)	1249.2 (1150.4, 1357.4)	20.9 (14.7, 27.1)
Turkmenistan	23114 (20834, 25648)	46517 (42266, 51634)	1180.4 (1062.1, 1298.9)	1178 (1075.5, 1286.1)	-0.2 (-3.9, 3.8)
Republic of Uzbekistan	127740 (118470, 138723)	384553 (361440, 413202)	1042.3 (965.9, 1129.3)	1721.9 (1633.5, 1826.6)	65.2 (56.6, 75.2)
Central Europe	1335444 (1233135, 1448390)	1509555 (1406290, 1627138)	935.3 (867.4, 1006.7)	700 (653.4, 751.2)	-25.2 (-27.2, -23.3)
Republic of Albania	17065 (15614, 18809)	30220 (27562, 33457)	777.4 (709.2, 858.6)	749.3 (690.4, 820.3)	-3.6 (-6.3, -0.8)
Bosnia and Herzegovina	30959 (28108, 34046)	44580 (40348, 49222)	788 (716.7, 864)	738.7 (671.9, 811.4)	-6.3 (-9.2, -3.1)
Republic of Bulgaria	116700 (108789, 126091)	116404 (108736, 125477)	1049.2 (990.3, 1118.8)	847.8 (795.8, 909.1)	-19.2 (-21.9, -16.1)
Republic of Croatia	48931 (45214, 53368)	52927 (49434, 56579)	850.1 (789.8, 916.7)	611.1 (572.4, 650.3)	-28.1 (-31.6, -25.4)
Czech Republic	135237 (126484, 144025)	160691 (148242, 173601)	1005.3 (944.9, 1068.5)	779.1 (718.9, 845.1)	-22.5 (-26.2, -18.5)
Hungary	150578 (139697, 164171)	143743 (132540, 156485)	1062 (987.8, 1151.2)	752 (697.8, 816.7)	-29.2 (-31.8, -26.1)
Montenegro	4824 (4355, 5374)	7560 (6815, 8544)	790.5 (714.5, 879.3)	806.6 (733.8, 903.4)	2 (-0.8, 4.8)
North Macedonia	16008 (14483, 17622)	26508 (23900, 29361)	892.9 (813.9, 977.9)	895 (818.5, 980.7)	0.2 (-3.2, 3.6)
Republic of Poland	360591 (316377, 410371)	386318 (355918, 417727)	853.9 (752.7, 965.2)	557.5 (516.9, 600.3)	-34.7 (-39.8, -29.2)
Romania	256354 (239135, 276125)	278115 (261535, 298194)	993 (934.7, 1059.2)	760.2 (714.9, 816.8)	-23.4 (-26.2, -20.7)
Republic of Serbia	102498 (95233, 109906)	144855 (134369, 157601)	974.7 (914.9, 1040.6)	901.6 (838.4, 981.5)	-7.5 (-11.8, -2.2)
Slovak Republic	55882 (51727, 60234)	67735 (62464, 73256)	952.5 (886.2, 1024.4)	730.3 (677, 788.4)	-23.3 (-25.8, -20.2)
Republic of Slovenia	18462 (16634, 20541)	27928 (25018, 31265)	763.1 (690.9, 847)	655.9 (591.9, 727.9)	-14.1 (-17.6, -10.6)
Central Latin America	622819 (567817, 683933)	1549589 (1397891, 1727219)	694.6 (630.3, 771.4)	620.1 (558.5, 689.3)	-10.7 (-12, -9.6)
Republic of Colombia	119971 (110197, 132442)	300821 (274738, 334143)	671.8 (616.7, 737.5)	539 (491.9, 598.1)	-19.8 (-22.4, -17.1)
Republic of Costa Rica	14724 (13314, 16324)	37160 (33231, 41958)	744.8 (666.2, 832.8)	687.5 (615.9, 771.4)	-7.7 (-10.5, -5.2)
Republic of El Salvador	22510 (20376, 24845)	40117 (36240, 44513)	648.1 (584.8, 720.1)	625 (561.7, 695.8)	-3.6 (-6.3, -0.8)
Republic of Guatemala	27118 (24486, 30000)	75119 (68172, 83816)	633.1 (578, 700.9)	632.8 (578.2, 702.1)	-0.1 (-3.6, 2.9)
Republic of Honduras	17349 (15507, 19306)	50013 (45101, 54932)	692.8 (622.8, 776.6)	736.1 (670.8, 807.7)	6.2 (1.9, 10.4)
United Mexican States	325922 (294549, 360111)	798458 (705368, 897139)	705.6 (631.8, 789.8)	636.9 (565.7, 714.5)	-9.7 (-11.1, -8.3)
Republic of Nicaragua	13907 (12536, 15319)	34396 (31072, 38101)	715 (643.5, 794.4)	662.9 (597.4, 739.5)	-7.3 (-10.7, -4.1)
Republic of Panama	11392 (10353, 12682)	28778 (25883, 32257)	693.6 (628.4, 773.1)	647.6 (582.5, 725.6)	-6.6 (-9.3, -4.1)
Bolivarian Republic of Venezuela	69926 (63867, 77202)	184727 (169173, 203845)	707.1 (641.7, 777.2)	620 (570.4, 680.7)	-12.3 (-15.2, -9.4)
Central Sub-Saharan Africa	239984 (215804, 265235)	570416 (509190, 630884)	853.2 (775.4, 937.9)	786.4 (721.7, 859.2)	-7.8 (-10.5, -5.5)
Republic of Angola	43627 (39163, 48334)	127483 (112646, 142159)	880.4 (792, 969.5)	791.3 (720.2, 874.1)	-10.1 (-13.4, -6.7)
Central African Republic	12431 (11102, 13896)	24352 (21493, 27106)	886.2 (789.8, 990.4)	859.9 (769.7, 959.9)	-3 (-5.6, -0.2)
Republic of the Congo	11758 (10564, 13048)	26944 (24202, 29968)	913.3 (820.3, 1011.1)	853.3 (765.8, 950.2)	-6.6 (-9.1, -4)
Democratic Republic of the Congo	164943 (147973, 183125)	376581 (336066, 417861)	840.6 (767.3, 921.2)	775.5 (712.5, 844.8)	-7.7 (-11.1, -4.7)
Republic of Equatorial Guinea	2024 (1816, 2240)	5719 (5085, 6409)	882.7 (793.3, 988.6)	797.1 (706.6, 903.4)	-9.7 (-12.5, -6.9)
Gabonese Republic	5201 (4653, 5814)	9337 (8346, 10354)	816.3 (732.4, 910.3)	799 (711, 890.7)	-2.1 (-5.2, 0.8)
East Asia	6501316 (5959780, 7129047)	16582690 (14941764, 18372360)	785.6 (719.7, 858.4)	809.3 (734.4, 888.7)	3 (0.8, 5.2)
People's Republic of China	6248590 (5727577, 6853739)	16039153 (14443325, 17780115)	783.9 (718.4, 857.6)	811.8 (736.1, 892)	3.6 (1.3, 5.8)
Democratic People's Republic of Korea	130274 (119503, 141471)	263609 (243181, 286046)	847.8 (778, 919.6)	845.4 (781.6, 914.7)	-0.3 (-3.9, 4)
Taiwan (Province of China)	122452 (111061, 134407)	279928 (251341, 313048)	817.1 (742.1, 899.3)	676.1 (610.6, 750.3)	-17.3 (-20.2, -14.3)
Eastern Europe	3084133 (2725612, 3457128)	3914587 (3418554, 4446707)	1177.9 (1049, 1316.1)	1135.6 (1000.2, 1279.3)	-3.6 (-5.9, -1.1)
Republic of Belarus	129309 (120204, 140349)	157859 (147479, 170692)	1033.3 (966, 1115.9)	997.9 (936.3, 1075.8)	-3.4 (-7.5, -0.1)
Republic of Estonia	20755 (19270, 22468)	23789 (20790, 27257)	1051.1 (980.5, 1132)	908.4 (798.8, 1034.6)	-13.6 (-21.3, -3.5)
Republic of Latvia	34143 (31410, 37096)	30802 (28204, 33805)	976.8 (903.5, 1057.8)	778.6 (712.5, 855.5)	-20.3 (-23.6, -17.2)
Republic of Lithuania	45009 (42182, 48345)	47169 (43685, 51195)	1013.1 (951, 1090)	812.3 (751.6, 882.7)	-19.8 (-23.8, -16.2)
Republic of Moldova	42153 (38658, 46002)	51445 (47126, 56196)	1066.5 (986.7, 1150.9)	873 (801.6, 950.6)	-18.1 (-21.1, -14.7)
Russian Federation	2034167 (1786246, 2295087)	2718586 (2366402, 3102561)	1212.2 (1071.7, 1365.3)	1160.7 (1018.9, 1312.7)	-4.2 (-6.8, -1.6)
Ukraine	778596 (684066, 878099)	884937 (764962, 1018392)	1153.1 (1018.3, 1296)	1156.2 (1012.7, 1316.9)	0.3 (-3.4, 3.7)
Eastern Sub-Saharan Africa	756878 (681693, 838703)	1681501 (1503975, 1866178)	782.8 (708.8, 860.2)	737.7 (670, 813.8)	-5.8 (-7.2, -4.5)
Republic of Burundi	24894 (22607, 27454)	50648 (44934, 57024)	852.6 (775, 937.9)	743.7 (672.5, 826.7)	-12.8 (-15.3, -10.2)
Union of the Comoros	1975 (1775, 2201)	4006 (3594, 4460)	802.7 (721.6, 894.9)	751.5 (669.9, 845.7)	-6.4 (-9.1, -3.7)
Republic of Djibouti	1514 (1351, 1703)	5881 (5261, 6598)	790.2 (707, 881.7)	793.6 (706.4, 896.4)	0.4 (-2.7, 3.3)
State of Eritrea	13488 (11937, 15136)	28174 (25276, 31436)	842 (762.5, 930.2)	762.9 (689.1, 846.9)	-9.4 (-12.1, -6.5)
Federal Democratic Republic of Ethiopia	189902 (171973, 210976)	395452 (352699, 444180)	763.7 (691.5, 844.8)	657.5 (590.6, 732.6)	-13.9 (-16.3, -11.6)
Republic of Kenya	85623 (76509, 95183)	212853 (189715, 237157)	766.4 (680.1, 861.4)	756.7 (672.9, 852.1)	-1.3 (-2.3, -0.2)
Republic of Madagascar	54431 (49588, 59857)	123654 (111247, 137407)	861.8 (786.6, 941.9)	834.8 (764.6, 912.6)	-3.1 (-5.6, -0.3)
Republic of Malawi	39716 (35611, 44187)	79754 (70462, 89234)	792.8 (714.5, 877.2)	774.7 (705.2, 863)	-2.3 (-5.4, 0.6)
Republic of Mozambique	60629 (54535, 67254)	128787 (114919, 143296)	822.8 (737.8, 906.2)	825.1 (748.7, 908.8)	0.3 (-3.1, 3.3)
Republic of Rwanda	31318 (28376, 34568)	56228 (50223, 62474)	869.3 (792, 952.7)	724.7 (655.7, 800.9)	-16.6 (-19.3, -14.3)
Federal Republic of Somalia	29101 (25978, 32470)	75887 (66863, 85412)	822.9 (738.5, 913.8)	780.7 (706.5, 862)	-5.1 (-8.1, -2.3)
Republic of South Sudan	23940 (21481, 26674)	37693 (33440, 42242)	741.7 (667.9, 825.2)	734 (657.5, 818.2)	-1 (-3.5, 1.6)
Republic of Uganda	66848 (59713, 74528)	153801 (136915, 172409)	768.3 (696, 847.9)	716.3 (652.8, 795.1)	-6.8 (-9.7, -4)
United Republic of Tanzania	101809 (91380, 112600)	251723 (226768, 277798)	725.5 (659.1, 803)	761.1 (696.6, 836.6)	4.9 (1.6, 8.6)
Republic of Zambia	31148 (27851, 34851)	75495 (67331, 84656)	792 (716.2, 881.4)	776.6 (705.6, 852.6)	-1.9 (-5, 0.9)
High-income Asia Pacific	1140579 (1066155, 1220532)	1818385 (1684621, 1958696)	576.2 (541.2, 614.8)	422.7 (395.2, 451.9)	-26.6 (-28.6, -24.8)
Brunei Darussalam	760 (709, 807)	1770 (1641, 1896)	674 (624.2, 723.5)	500.4 (464.2, 539.7)	-25.8 (-28, -23.3)
Japan	896727 (834584, 962749)	1399673 (1289572, 1512181)	540.1 (505.1, 578.4)	424.2 (395.9, 454.3)	-21.4 (-23.6, -19.3)
Republic of Korea	228140 (214240, 243480)	380688 (355710, 409284)	798.6 (752.4, 850.1)	423.5 (399, 452.4)	-47 (-49.2, -44.9)
Republic of Singapore	14952 (13773, 16185)	36254 (32996, 40136)	671.9 (616.4, 732.9)	428.7 (389.8, 472.1)	-36.2 (-38.4, -34)
High-income North America	3435273 (3053923, 3822225)	4293532 (4048636, 4558009)	986.6 (881.2, 1101.4)	670.2 (635.1, 708.8)	-32.1 (-37, -27.2)
Canada	306643 (285774, 332016)	446471 (412942, 486276)	944.7 (881.7, 1020.8)	638.4 (592, 694.3)	-32.4 (-35.1, -29.6)
Greenland	309 (285, 335)	504 (458, 556)	943.8 (867.6, 1021.5)	747.5 (685.1, 817.6)	-20.8 (-23.1, -18.4)
United States of America	3128242 (2768304, 3503017)	3846490 (3627397, 4079395)	991.8 (881, 1113.7)	673.9 (638.8, 712.3)	-32.1 (-37.5, -26.6)
North Africa and the Middle East	2293414 (2062501, 2581476)	5660724 (5097761, 6383725)	1336.5 (1212.7, 1504.3)	1232.3 (1117.9, 1378.3)	-7.8 (-9.7, -5.8)
Islamic Republic of Afghanistan	105721 (95044, 119582)	153468 (137323, 169849)	1563.5 (1416.2, 1753)	1346.9 (1215.6, 1512.5)	-13.9 (-18.1, -8.7)
People's Democratic Republic of Algeria	177773 (158849, 203333)	428016 (383679, 485882)	1482.4 (1340.6, 1672.8)	1210.5 (1095.9, 1357.3)	-18.3 (-22.5, -14)
Kingdom of Bahrain	2499 (2102, 2974)	12805 (10322, 15944)	1377 (1138.9, 1642.9)	1284.5 (1057.5, 1553)	-6.7 (-11, -3.1)
Arab Republic of Egypt	387391 (349382, 432242)	924213 (828860, 1022544)	1420 (1288.7, 1570.3)	1441.1 (1301.8, 1590.9)	1.5 (-4.9, 8.5)
Islamic Republic of Iran	355774 (301352, 420979)	923522 (782697, 1088275)	1337.4 (1121.7, 1559.6)	1197.1 (1008.8, 1398.4)	-10.5 (-12.1, -8.8)
Republic of Iraq	123556 (111584, 137878)	354990 (318470, 399941)	1440.1 (1293.6, 1624.8)	1455.3 (1320, 1620.4)	1.1 (-3.9, 6.6)
Hashemite Kingdom of Jordan	18136 (15394, 21400)	96120 (81239, 114894)	1347 (1145.5, 1571.6)	1249.5 (1067.8, 1467.6)	-7.2 (-11.5, -3.2)
State of Kuwait	8826 (7302, 10579)	42798 (35413, 51761)	1360.6 (1116.8, 1655.5)	1336 (1108.7, 1604.3)	-1.8 (-6.6, 3.4)
Lebanese Republic	27568 (23451, 32243)	74515 (64102, 85145)	1313.4 (1125.7, 1505.1)	1238.3 (1059.5, 1428.4)	-5.7 (-10.1, -1.2)
State of Libya	24092 (20732, 28132)	67336 (57907, 78283)	1178.1 (1000.4, 1380.6)	1249.9 (1076, 1448.3)	6.1 (0.8, 11.2)
Kingdom of Morocco	200345 (181169, 223397)	458284 (412957, 512733)	1369.2 (1236.8, 1532)	1362.5 (1240.8, 1513.1)	-0.5 (-5.8, 4.5)
Sultanate of Oman	9139 (7910, 10473)	28740 (24193, 34172)	1320.8 (1129.1, 1527.4)	1333.2 (1133.8, 1564.9)	0.9 (-4, 5.8)
Palestine	12063 (10395, 13945)	34287 (29309, 40196)	1331.3 (1137.8, 1543.6)	1309.2 (1127.2, 1524.8)	-1.7 (-5.4, 1.9)
State of Qatar	1939 (1620, 2320)	16434 (13220, 20056)	1394.5 (1155.5, 1671.6)	1312.8 (1075.8, 1587.5)	-5.9 (-9.4, -2.3)
Kingdom of Saudi Arabia	70666 (61531, 81442)	266935 (231937, 309504)	1184.1 (1027.4, 1365)	1218.1 (1076.5, 1379.6)	2.9 (-2.3, 8.5)
Republic of Sudan	137550 (123162, 154163)	278907 (252231, 312105)	1436.5 (1284.8, 1618.3)	1337 (1206.4, 1495.4)	-6.9 (-11.3, -2.4)
Syrian Arab Republic	81790 (73540, 92727)	185427 (165398, 212313)	1467.4 (1323.2, 1656.3)	1480.4 (1334.6, 1657)	0.9 (-4.2, 6)
Republic of Tunisia	56032 (47630, 65542)	148809 (129495, 173415)	1155.8 (996.5, 1336)	1138.7 (1000.8, 1311.2)	-1.5 (-6.7, 3.3)
Republic of Turkey	408497 (369327, 460163)	872143 (776615, 992314)	1163.3 (1055.6, 1300.7)	939.2 (837.9, 1058.4)	-19.3 (-24.6, -13.6)
United Arab Emirates	8807 (7520, 10267)	85873 (69396, 104217)	1516.4 (1265.8, 1796)	1464.5 (1235.1, 1723.2)	-3.4 (-7.5, 1.6)
Republic of Yemen	73997 (65832, 83114)	201823 (180652, 226895)	1397.5 (1244.8, 1567.5)	1318.7 (1187.1, 1480.5)	-5.6 (-10.6, -0.1)
Oceania	26088 (23695, 28857)	63908 (57780, 70688)	821.1 (735.9, 920.8)	807.5 (724.6, 906.9)	-1.7 (-3.5, 0.2)
Independent State of Samoa	847 (767, 938)	1353 (1213, 1511)	917.7 (821.7, 1024.3)	908.7 (809, 1031.9)	-1 (-3.3, 1.4)
American Samoa	211 (191, 232)	403 (360, 454)	858 (765.7, 957.9)	881.5 (785.6, 993.9)	2.7 (0, 5.5)
Cook Islands	89 (80, 100)	190 (166, 217)	735.2 (654, 829.3)	754.6 (665.3, 855.1)	2.6 (-0.3, 5.9)
Republic of Fiji	3441 (3101, 3826)	6657 (5930, 7467)	920.5 (818.1, 1032.8)	910.5 (815, 1021)	-1.1 (-3.8, 1.6)
Guam	578 (523, 639)	1622 (1435, 1852)	726.1 (650.4, 814.2)	788.7 (700.2, 897)	8.6 (5.5, 11.7)
Republic of Kiribati	427 (392, 468)	779 (707, 857)	1060.6 (963.4, 1178.4)	1036.8 (933.5, 1157.6)	-2.2 (-4.7, 0.3)
Republic of the Marshall Islands	179 (163, 197)	323 (292, 355)	910.6 (823.4, 1019.1)	921.3 (827.5, 1025.3)	1.2 (-1.4, 3.8)
Federated States of Micronesia	513 (465, 567)	678 (613, 756)	952.1 (854.6, 1060.3)	940.2 (843.7, 1046.6)	-1.3 (-3.5, 1.3)
Republic of Nauru	41 (38, 46)	51 (46, 57)	906.3 (811.3, 1014.9)	875.1 (774.1, 990.8)	-3.4 (-6.4, -0.4)
Republic of Niue	19 (17, 21)	17 (15, 20)	837.1 (746.3, 933.2)	822.3 (734.9, 927.7)	-1.8 (-4.2, 1)
Northern Mariana Islands	163 (147, 180)	392 (345, 449)	808.5 (720.4, 909.8)	822 (724.1, 934.8)	1.7 (-1.5, 4.6)
Republic of Palau	77 (69, 85)	174 (155, 197)	808 (726.8, 900)	834.7 (746.1, 945.5)	3.3 (0, 6.4)
Independent State of Papua New Guinea	15018 (13588, 16665)	41827 (37886, 46261)	764.7 (683.6, 860)	754.9 (677.2, 849.2)	-1.3 (-4, 1.6)
Solomon Islands	1569 (1441, 1709)	3931 (3601, 4289)	1039.8 (942.1, 1153.3)	1065.9 (965.3, 1183.1)	2.5 (0, 5.1)
Tokelau	10 (9, 11)	11 (10, 13)	754.2 (674.7, 846.5)	755.9 (665.8, 859.4)	0.2 (-3.2, 3.2)
Kingdom of Tonga	505 (454, 564)	720 (643, 808)	832 (741.5, 939.6)	855.7 (760, 968.7)	2.9 (0.4, 5.7)
Tuvalu	52 (47, 58)	83 (74, 93)	834.1 (751.3, 931.9)	831.1 (741.9, 938.4)	-0.4 (-3.2, 2.7)
Republic of Vanuatu	684 (620, 752)	1828 (1660, 2022)	966.5 (863.5, 1083.5)	998.9 (892.9, 1121.6)	3.3 (0.3, 6.3)
South Asia	5735469 (5054475, 6512753)	13568431 (11969604, 15355222)	957 (838.1, 1087.4)	912.2 (805.6, 1032.6)	-4.7 (-5.8, -3.6)
People's Republic of Bangladesh	467609 (426063, 513611)	1165216 (1067985, 1282289)	902.6 (823.7, 995.1)	839.1 (770.1, 917.3)	-7 (-11.2, -2.8)
Kingdom of Bhutan	2458 (2126, 2868)	5492 (4683, 6421)	914.2 (777, 1071.6)	893 (761.5, 1048.3)	-2.3 (-5.8, 1)
Republic of India	4549273 (3985465, 5206859)	10778160 (9415354, 12273924)	954.2 (831, 1089.7)	905.2 (791.7, 1031.3)	-5.1 (-6.2, -4.1)
Federal Democratic Republic of Nepal	87161 (76787, 99295)	195886 (175366, 220311)	878.7 (771, 999.1)	833.1 (746.7, 931.6)	-5.2 (-9.4, -0.7)
Islamic Republic of Pakistan	628968 (557541, 706496)	1423677 (1256930, 1608264)	1039.7 (906.6, 1177.1)	1066.2 (931.9, 1212.4)	2.6 (0.4, 4.9)
Southeast Asia	1825627 (1702564, 1967343)	4308638 (3998289, 4666767)	699.3 (649.8, 758.4)	675.1 (627.4, 730.3)	-3.5 (-4.7, -2.2)
Kingdom of Cambodia	34617 (32054, 37555)	85900 (79634, 92848)	704.6 (651.3, 764.8)	706.8 (659.2, 760.2)	0.3 (-2.7, 3.9)
Republic of Indonesia	710354 (655103, 770122)	1741176 (1595929, 1900154)	717.8 (659.2, 782.2)	764 (699.4, 834.5)	6.4 (4.8, 8)
Lao People's Democratic Republic	17625 (16240, 19137)	34953 (32385, 37807)	791.2 (731.3, 860.7)	730.7 (676.4, 792.1)	-7.7 (-10.3, -4.9)
Malaysia	80931 (75320, 87143)	210378 (195372, 228094)	806.4 (746.9, 876.1)	752.2 (699.3, 812.3)	-6.7 (-10, -3.4)
Republic of Maldives	745 (685, 805)	2197 (2013, 2382)	737.8 (674.8, 805.2)	611.1 (553.6, 675.2)	-17.2 (-19.6, -14.6)
Republic of Mauritius	5723 (5250, 6247)	10749 (9743, 11992)	776.8 (709.9, 847.6)	624.7 (569.2, 689.4)	-19.6 (-22.1, -17)
Republic of the Union of Myanmar	200262 (185723, 217259)	337485 (314883, 363217)	825.9 (770.2, 889.2)	705.2 (661.8, 757.4)	-14.6 (-17.4, -12)
Republic of the Philippines	195612 (179712, 214616)	509136 (472200, 552124)	602.6 (547.4, 668.5)	603.3 (555.6, 656.4)	0.1 (-3, 2.8)
Republic of Seychelles	390 (357, 429)	715 (650, 791)	661.1 (603.6, 727.9)	640.1 (581.8, 709)	-3.2 (-5.4, -0.6)
Democratic Socialist Republic of Sri Lanka	67974 (62786, 73949)	159631 (146999, 173450)	661.6 (606.8, 720.8)	614.5 (570.2, 665.7)	-7.1 (-9.9, -4.2)
Kingdom of Thailand	245611 (229463, 264903)	558997 (520071, 608408)	664.6 (624.1, 719.3)	552.2 (516.1, 595.4)	-16.9 (-18.9, -14.8)
Democratic Republic of Timor-Leste	2138 (1958, 2327)	5999 (5486, 6576)	635 (579.6, 699.1)	663.3 (604.3, 728)	4.5 (1.4, 7.1)
Socialist Republic of Viet Nam	261006 (244156, 280861)	645311 (603236, 693316)	655.6 (613.2, 703.4)	670 (629.8, 715)	2.2 (-1, 5.9)
Southern Latin America	351406 (328339, 377804)	484587 (454629, 520245)	770 (721.9, 826.2)	575.2 (540.1, 617.2)	-25.3 (-27.3, -23.2)
Eastern Republic of Uruguay	26559 (24405, 28733)	30725 (28129, 33734)	694.5 (641.7, 749.2)	566.2 (520.2, 620.9)	-18.5 (-21.4, -15.8)
Argentine Republic	264664 (247684, 285402)	342036 (318177, 370019)	834.3 (783.7, 897.7)	631.9 (587.9, 684.8)	-24.3 (-26.7, -21.8)
Republic of Chile	60167 (55941, 65015)	111799 (105251, 119350)	605.4 (561.7, 654.7)	443.2 (418.2, 472.7)	-26.8 (-30.7, -22.8)
Southern Sub-Saharan Africa	272173 (244832, 301425)	489804 (440661, 546039)	864.6 (773.9, 965.8)	815.5 (732.5, 910.4)	-5.7 (-7.1, -4.2)
Republic of Botswana	5705 (5111, 6344)	13477 (12143, 14965)	851 (764.5, 949.4)	865.5 (773.7, 964.3)	1.7 (-1.3, 4.7)
Kingdom of Eswatini	2980 (2657, 3328)	5240 (4703, 5808)	792.6 (710, 883.4)	826.8 (738.6, 925.3)	4.3 (1.2, 7.2)
Kingdom of Lesotho	7056 (6310, 7826)	9662 (8664, 10713)	731.9 (655.1, 815.7)	829.2 (749.3, 920.3)	13.3 (10, 16.3)
Republic of Namibia	6389 (5736, 7088)	11939 (10706, 13295)	854.6 (769.5, 955.8)	790.9 (708.8, 881.7)	-7.5 (-10.3, -4.8)
Republic of South Africa	211079 (189973, 234480)	382412 (342670, 430376)	895.9 (800.3, 1003.3)	816.4 (731.4, 912.9)	-8.9 (-10.5, -7.3)
Republic of Zimbabwe	38966 (34787, 43241)	67075 (60158, 74386)	745.6 (671.8, 831.7)	814.2 (735.4, 897.2)	9.2 (5.4, 12.9)
Tropical Latin America	698032 (646962, 758025)	1358155 (1252037, 1473102)	671.3 (624.6, 724.5)	547.1 (505, 592.5)	-18.5 (-20.3, -16.6)
Federative Republic of Brazil	682363 (632348, 741093)	1323880 (1220719, 1436092)	673 (626.1, 726.3)	546.9 (504.9, 592.1)	-18.7 (-20.6, -16.9)
Republic of Paraguay	15669 (14327, 17048)	34274 (31528, 37432)	602.5 (555.8, 659.3)	558.7 (512.8, 610.3)	-7.3 (-9.5, -4.9)
Western Europe	4557942 (4280054, 4869798)	5102835 (4795711, 5455074)	800.6 (757.2, 854.4)	576.7 (542, 619.3)	-28 (-29.6, -26.4)
Principality of Andorra	338 (307, 373)	757 (686, 838)	588.9 (535.3, 647.5)	501 (456.3, 554.3)	-14.9 (-16.8, -12.8)
Republic of Austria	85835 (81283, 90984)	122031 (116153, 129637)	736.1 (699.7, 777.1)	701.1 (663.9, 745.8)	-4.8 (-8.1, -0.8)
Kingdom of Belgium	115940 (108126, 125641)	123195 (114166, 133819)	771.3 (719.7, 835.9)	549.7 (509.8, 597.1)	-28.7 (-32.3, -24.9)
Republic of Cyprus	5128 (4711, 5612)	9841 (9100, 10715)	626.2 (577.6, 681.6)	486.9 (450.9, 526.6)	-22.2 (-24.6, -19.8)
Kingdom of Denmark	53256 (49837, 57010)	62318 (57264, 67780)	666.7 (627.1, 712.1)	552.9 (511.3, 599.5)	-17.1 (-21, -12.6)
Republic of Finland	72799 (68060, 78240)	79407 (73650, 85753)	1042.4 (976.2, 1120.1)	652 (604, 704.6)	-37.4 (-40.8, -34)
French Republic	570641 (532976, 612203)	716849 (664529, 776256)	698.7 (656, 745)	533.7 (494.5, 580.2)	-23.6 (-27.2, -19.6)
Federal Republic of Germany	1208411 (1143814, 1278359)	1233101 (1161365, 1314707)	966.5 (917.5, 1020.3)	673.4 (633.7, 720.2)	-30.3 (-33.8, -26.6)
Hellenic Republic	107091 (100144, 115979)	122897 (115496, 132239)	719.4 (673.7, 775.3)	526.3 (493, 566.3)	-26.8 (-29.4, -24.2)
Republic of Iceland	2048 (1859, 2261)	3150 (2865, 3472)	725.9 (659.1, 799)	560.8 (512.2, 615.1)	-22.7 (-26.5, -19.2)
Ireland	32953 (30363, 36023)	39516 (36009, 43442)	809.7 (750, 882.5)	511.4 (467, 560.8)	-36.8 (-39.6, -34.4)
State of Israel	35792 (32805, 39227)	65365 (60275, 70938)	740.5 (683.5, 805)	540 (498.9, 586.4)	-27.1 (-29.9, -24.1)
Republic of Italy	699729 (638117, 764176)	810477 (754270, 873104)	807.3 (740.5, 878.2)	606.9 (567.5, 651.6)	-24.8 (-27.8, -21.9)
Grand Duchy of Luxembourg	3396 (3145, 3672)	5377 (4973, 5807)	633 (587.7, 682.6)	522.9 (484.5, 564.8)	-17.4 (-19.8, -14.9)
Republic of Malta	2575 (2366, 2807)	4280 (3923, 4672)	608.3 (560, 660.9)	461.5 (426, 499.4)	-24.1 (-26.9, -21.6)
Principality of Monaco	429 (392, 471)	473 (429, 524)	637.8 (586.2, 695.5)	519.1 (474.3, 575)	-18.6 (-20.7, -16.6)
Kingdom of the Netherlands	158038 (147352, 169780)	185253 (170861, 202243)	799.8 (746.4, 861.1)	549.5 (508.2, 597.9)	-31.3 (-34.3, -28.1)
Kingdom of Norway	56878 (51967, 62434)	60406 (55408, 65786)	848.8 (779.4, 921.2)	623.5 (575.1, 677.7)	-26.5 (-28.4, -24.7)
Portuguese Republic	93725 (87052, 101395)	98432 (91958, 105411)	692.9 (646.3, 745.2)	415.7 (390.1, 448.4)	-40 (-42.4, -37.5)
Republic of San Marino	213 (194, 234)	365 (331, 403)	618.3 (565, 676.2)	517.4 (471.7, 568.1)	-16.3 (-18.4, -14.2)
Kingdom of Spain	381598 (357691, 410285)	475967 (449473, 506557)	713.2 (672.6, 762.8)	514.5 (485.4, 548.8)	-27.9 (-31, -25.1)
Kingdom of Sweden	115354 (108469, 123702)	143524 (129190, 158822)	768.1 (724.9, 818.5)	695 (630.6, 763.9)	-9.5 (-15.5, -3.1)
Swiss Confederation	68559 (64182, 73596)	86856 (80928, 93880)	666.3 (624.1, 712.6)	496 (459.5, 537.3)	-25.6 (-28.7, -22.6)
United Kingdom of Great Britain and Northern Ireland	683470 (622013, 752170)	648507 (602407, 699222)	771.3 (705.2, 844.9)	526.6 (489.7, 566.8)	-31.7 (-34.3, -29.5)
Western Sub-Saharan Africa	805740 (731481, 887909)	1878727 (1699128, 2071910)	785.2 (705, 874.6)	765.8 (689, 848.5)	-2.5 (-4.1, -1.1)
Republic of Benin	19280 (17447, 21203)	49258 (44100, 54360)	789.4 (707.8, 885.1)	749.4 (672.9, 838.1)	-5.1 (-8.3, -2)
Burkina Faso	35382 (31877, 39474)	79207 (71573, 87566)	695.3 (623.3, 775.4)	683.9 (613, 761.4)	-1.6 (-5.5, 2.3)
Republic of Cabo Verde	1885 (1675, 2143)	3467 (3081, 3914)	730.6 (645.7, 834.8)	753.2 (668, 859.6)	3.1 (-0.3, 6.7)
Republic of Cameroon	37566 (34051, 41478)	116593 (105708, 128555)	709.7 (640.5, 785.2)	739.1 (671.9, 814.1)	4.2 (-0.2, 8.1)
Republic of Chad	25686 (23229, 28481)	59824 (53774, 65855)	779.1 (702.5, 867.5)	772.4 (693.1, 857.8)	-0.9 (-4.4, 2.4)
Republic of Côte d'Ivoire	43057 (39008, 47431)	108576 (98219, 119275)	827.2 (744.4, 916.3)	787.6 (710.7, 867.5)	-4.8 (-8.4, -1.3)
Republic of the Gambia	3618 (3246, 4015)	9846 (8850, 10966)	816.5 (717.6, 930.2)	820.3 (730.6, 927.8)	0.5 (-3.2, 3.4)
Republic of Ghana	65176 (59527, 70956)	161240 (147776, 176611)	879.9 (798.7, 968.7)	834.8 (766, 911.4)	-5.1 (-8.2, -2.4)
Republic of Guinea-Bissau	4080 (3684, 4537)	7703 (6935, 8498)	852.9 (758.9, 962.8)	832.4 (743.8, 936.5)	-2.4 (-5.1, 0.5)
Republic of Guinea	26871 (24481, 29638)	52093 (47329, 57451)	746.9 (673.9, 826.7)	770.2 (699.7, 850.2)	3.1 (-0.5, 6.6)
Republic of Liberia	10774 (9715, 11914)	20849 (18708, 23193)	805.3 (714.3, 901.1)	774.5 (686.3, 870.4)	-3.8 (-6.7, -1)
Republic of Mali	34830 (31501, 38880)	82605 (73699, 91378)	759.1 (682.3, 846.7)	721 (651.2, 805.6)	-5 (-8.1, -2.1)
Islamic Republic of Mauritania	9529 (8586, 10601)	19083 (17158, 21395)	837.6 (747.1, 940.1)	763.9 (679.1, 864.4)	-8.8 (-11.4, -5.8)
Republic of the Niger	27504 (24835, 30370)	80657 (72201, 89100)	771.9 (690.2, 862.2)	728.2 (658.7, 811.8)	-5.7 (-9.4, -2.3)
Federal Republic of Nigeria	394291 (355832, 436046)	883956 (792082, 979330)	780.6 (697.7, 871.8)	766.6 (682.9, 862.3)	-1.8 (-3.4, -0.3)
Democratic Republic of São Tomé and Príncipe	586 (527, 657)	1040 (938, 1150)	798.6 (711.8, 904.8)	814.5 (724.1, 921.9)	2 (-0.5, 4.8)
Republic of Senegal	32285 (29139, 35753)	70386 (63449, 77577)	832.8 (742.2, 931.9)	777.6 (699.2, 860.9)	-6.6 (-10, -3.5)
Republic of Sierra Leone	20086 (18178, 22105)	37124 (33458, 41042)	852.5 (760.8, 955.1)	797.4 (715.5, 892.6)	-6.5 (-9.1, -3.7)
Togolese Republic	13226 (11919, 14498)	35199 (31666, 39216)	823.7 (736.3, 922)	788.8 (710.1, 880.3)	-4.2 (-7.6, -1)

**Table 3:** The number of total deaths of cardiovascular disease and their ASDR for 204 countries worldwide in 1990 and 2021 and the percentage change in ASDR from 1990 to 2021 were calculated ASDR: age-standardized death rates (per 100,000); 95% UI: 95% uncertainty intervals The number of total deaths=total deaths×1 Percentage change in the ASDR per 100,000=(the ASDR in 2021-the ASDR in 1990)/the ASDR in 1990

	Total deaths (95% UI)		ASDR per 100,000 (95% UI)		Percentage change in the ASDR per 100,000
	1990	2021	1990	2021	
Global	12330009 (11626405, 12787472)	19414853 (17775807, 20668512)	358.1 (333.7, 372.6)	235.2 (214.6, 250.5)	-34.3 (-37.8, -30.7)
Andean Latin America	37599 (34861, 40794)	65684 (56153, 77453)	196.8 (182.5, 212.8)	115.4 (98.7, 135.8)	-41.4 (-50.5, -31.4)
Plurinational State of Bolivia	8730 (7327, 10872)	14174 (10843, 19020)	309.7 (262.9, 378)	183.4 (142, 244.3)	-40.8 (-51.5, -24.8)
Republic of Ecuador	10207 (9781, 10514)	21306 (17545, 25848)	215 (204.7, 222.2)	143.4 (119.1, 172.2)	-33.3 (-44.1, -20.8)
Republic of Peru	18661 (16554, 20980)	30204 (24016, 37316)	163.5 (144.6, 183.6)	89.6 (71.4, 110.7)	-45.2 (-57.9, -30.5)
Australasia	62954 (58249, 65359)	58243 (49260, 62867)	280 (256.8, 291.9)	94.2 (80.8, 101.2)	-66.4 (-68.6, -65)
Australia	51657 (47830, 53668)	47254 (39856, 51110)	277.1 (254.3, 288.8)	89.7 (76.8, 96.4)	-67.6 (-69.9, -66.3)
New Zealand	11297 (10464, 11759)	10989 (9387, 11830)	294.6 (270.2, 307.6)	119.1 (102.6, 127.8)	-59.6 (-62.4, -57.8)
Caribbean	82020 (78133, 84989)	124209 (110467, 138460)	342 (324.2, 354.2)	228.4 (203.3, 254.9)	-33.2 (-40.2, -25.8)
Antigua and Barbuda	179 (166, 189)	203 (192, 213)	313.1 (291.8, 331.6)	219.2 (206, 230.4)	-30 (-34, -25.6)
Commonwealth of the Bahamas	443 (414, 472)	834 (695, 1018)	307.3 (284.4, 326.9)	228.4 (191.3, 275.9)	-25.7 (-38.7, -9.7)
Barbados	868 (813, 908)	933 (778, 1107)	288.4 (269.9, 301.4)	181 (151.1, 214.7)	-37.2 (-48.3, -25.2)
Belize	212 (199, 223)	450 (400, 498)	225.9 (212.3, 237.7)	167.2 (148.1, 185.1)	-26 (-33.9, -18.2)
Bermuda	215 (204, 225)	211 (180, 250)	378.4 (356.7, 396.1)	139.1 (119.7, 165.3)	-63.2 (-68, -56.2)
Republic of Cuba	30651 (29328, 31388)	41280 (36208, 46169)	320 (304.3, 328.3)	198.3 (173.8, 221.5)	-38 (-45, -30.7)
Commonwealth of Dominica	214 (202, 225)	212 (191, 245)	374 (352.5, 392.8)	276.3 (250, 317.2)	-26.1 (-33.8, -15.2)
Dominican Republic	9028 (8010, 10064)	24509 (20033, 30077)	284.2 (253.4, 315.2)	251.4 (205.5, 307.8)	-11.6 (-29.8, 12.2)
Grenada	285 (262, 309)	256 (226, 284)	367.3 (337.8, 398)	260.6 (229.8, 286.2)	-29.1 (-38, -20.6)
Republic of Guyana	2109 (1920, 2302)	2126 (1701, 2638)	611.3 (558.1, 665.2)	378.7 (307.8, 461.8)	-38.1 (-51.4, -22.8)
Republic of Haiti	17022 (14724, 19527)	27803 (21259, 36230)	617.7 (539.6, 702.2)	466.4 (362.3, 594.9)	-24.5 (-41.7, -2.6)
Jamaica	4723 (4444, 4910)	6394 (5148, 7971)	254.7 (239.5, 264.8)	192 (154.2, 240.1)	-24.6 (-39, -6.5)
Puerto Rico	8284 (7865, 8563)	8163 (6634, 9464)	247.2 (233.3, 256)	96.2 (79.6, 111.3)	-61.1 (-67.4, -55.4)
Saint Kitts and Nevis	190 (180, 199)	150 (129, 170)	530 (501.3, 553.9)	278 (241.9, 308.2)	-47.6 (-54.3, -41.7)
Saint Lucia	327 (312, 341)	474 (398, 549)	448.7 (424.7, 466.2)	207.9 (173.3, 240.3)	-53.7 (-61, -46.6)
Saint Vincent and the Grenadines	260 (245, 274)	351 (316, 389)	396.1 (371.4, 417.6)	277.1 (250.7, 306.5)	-30 (-36.9, -22.5)
Republic of Suriname	836 (776, 885)	1339 (1050, 1635)	351.1 (324.7, 371)	222.2 (174.2, 271.5)	-36.7 (-50.5, -21.7)
Republic of Trinidad and Tobago	3155 (3035, 3250)	4021 (3165, 5016)	443.8 (423.4, 457.7)	217.1 (171.6, 270)	-51.1 (-61, -39.7)
United States Virgin Islands	240 (207, 269)	295 (241, 356)	361.4 (316.4, 401)	176.2 (144.3, 211.6)	-51.2 (-60.1, -40.6)
Central Asia	214255 (203736, 220441)	293313 (266420, 320330)	512.9 (484.2, 529.8)	436.1 (395.7, 474.5)	-15 (-21.7, -8)
Republic of Armenia	10810 (10231, 11220)	13535 (12102, 14992)	469 (440, 488.1)	314.8 (281, 348.5)	-32.9 (-38.5, -26.7)
Republic of Azerbaijan	23003 (21569, 24332)	35723 (31654, 39800)	539.6 (501.6, 569.4)	449.6 (401.1, 498.4)	-16.7 (-24.6, -7.5)
Georgia	33204 (31669, 34242)	22784 (20473, 24951)	583.1 (553.7, 602.5)	355.2 (319.9, 387.9)	-39.1 (-44.1, -33.9)
Republic of Kazakhstan	55977 (52827, 58985)	65091 (59485, 71178)	506.2 (474.9, 533.5)	461.4 (420.3, 501.6)	-8.9 (-17, -0.7)
Kyrgyz Republic	13099 (12116, 14074)	15679 (13223, 18086)	492.6 (454.7, 527.5)	404.2 (344.3, 463.4)	-17.9 (-29.5, -5.1)
Mongolia	5274 (4828, 5754)	6905 (6175, 7619)	585.7 (536.4, 634.2)	390.3 (346.6, 430.3)	-33.4 (-40.8, -24.8)
Republic of Tajikistan	12777 (11781, 13731)	17364 (14978, 19635)	519.6 (477, 557.6)	414.5 (356.4, 467.1)	-20.2 (-31.1, -9.3)
Turkmenistan	9596 (9241, 9852)	18841 (15329, 23236)	589.7 (559.9, 607.4)	552.2 (453.6, 672.8)	-6.4 (-23.5, 14.8)
Republic of Uzbekistan	50515 (47558, 52168)	97390 (84880, 110365)	475.2 (445.3, 492)	478.5 (417.3, 539.4)	0.7 (-11.6, 13.1)
Central Europe	723472 (695750, 737744)	682174 (621708, 724976)	542.8 (516.1, 555.8)	288 (262.8, 306.1)	-46.9 (-49.9, -44.1)
Republic of Albania	7195 (6737, 7498)	14053 (12122, 16166)	428.5 (399.2, 446.7)	345.2 (299.2, 396.8)	-19.4 (-30.2, -8.4)
Bosnia and Herzegovina	13799 (13040, 14359)	18769 (15476, 21612)	413.6 (386.4, 432.1)	294.6 (241.9, 340.2)	-28.8 (-40.4, -18.4)
Republic of Bulgaria	70055 (67655, 72286)	75617 (67014, 84127)	784.4 (752.7, 807.3)	530.3 (471.8, 589.1)	-32.4 (-39.4, -25.1)
Republic of Croatia	27939 (26684, 28863)	23393 (20722, 25530)	542.8 (514.9, 561.3)	236.7 (209.4, 258.1)	-56.4 (-60, -52.6)
Czech Republic	70630 (67457, 72491)	46794 (40858, 51444)	526.2 (499.8, 540.8)	200.8 (175.6, 221)	-61.8 (-65.5, -58.4)
Hungary	73830 (70803, 75786)	57964 (51068, 63709)	544.3 (518.2, 560.4)	272.7 (240.5, 300.1)	-49.9 (-54.8, -45.4)
Montenegro	2353 (2171, 2495)	4327 (3936, 4757)	408.2 (375, 432.9)	512.8 (466.6, 562.4)	25.6 (14.5, 38.3)
North Macedonia	9219 (8743, 9587)	13288 (11439, 15226)	569.4 (539.4, 593)	560.2 (491.4, 627.4)	-1.6 (-13.4, 9.8)
Republic of Poland	201973 (192540, 206796)	168854 (149611, 183007)	498.3 (470.9, 511.7)	217.8 (194, 236)	-56.3 (-59.6, -53.2)
Romania	139226 (134082, 142069)	146016 (132456, 159320)	613 (585.1, 627.8)	359.5 (326.6, 392.4)	-41.4 (-46.5, -36.6)
Republic of Serbia	58483 (55936, 60530)	68868 (60551, 78048)	719.7 (687.7, 744.6)	397.9 (349.3, 450.8)	-44.7 (-51, -37.7)
Slovak Republic	29134 (27751, 30176)	27057 (23757, 30077)	509.6 (482.2, 528.6)	285.3 (250.3, 317.1)	-44 (-49.7, -38.2)
Republic of Slovenia	8067 (7551, 8427)	7245 (6078, 8110)	333.5 (310.6, 349.3)	131.9 (111.7, 147.7)	-60.4 (-64.7, -56.5)
Central Latin America	165207 (157754, 168746)	389477 (347068, 430749)	230.6 (217.5, 236.6)	163.7 (145.6, 180.7)	-29 (-35.3, -22.3)
Republic of Colombia	42075 (40060, 43156)	74747 (62393, 87648)	271.1 (255.1, 279.2)	132.4 (110.9, 155.4)	-51.2 (-58.2, -43.4)
Republic of Costa Rica	3369 (3113, 3532)	6130 (5324, 6838)	205.1 (188.3, 215.4)	108.9 (95.1, 121)	-46.9 (-52.6, -41.5)
Republic of El Salvador	5802 (5433, 6089)	9641 (7913, 11495)	198.5 (185.4, 208.5)	143 (117.5, 170.7)	-28 (-39.9, -14.7)
Republic of Guatemala	6379 (6166, 6536)	14509 (12661, 16432)	240.8 (230.9, 247.2)	155.2 (136.3, 174.6)	-35.6 (-42.9, -28.1)
Republic of Honduras	4449 (3915, 4986)	17452 (15256, 20595)	248.4 (218.3, 277.9)	336.4 (294.3, 391.9)	35.5 (15.6, 62.8)
United Mexican States	72046 (69579, 73360)	189716 (168616, 209944)	209.7 (200.3, 214.2)	165.9 (148, 183.3)	-20.9 (-28.2, -13.1)
Republic of Nicaragua	2439 (2260, 2577)	5685 (4852, 6702)	176.2 (162, 186.8)	134.7 (114.9, 157.3)	-23.5 (-33.9, -10.7)
Republic of Panama	2789 (2604, 2909)	5598 (4368, 6678)	201.6 (186.9, 210.8)	122.2 (96, 146.1)	-39.4 (-51.2, -28.6)
Bolivarian Republic of Venezuela	25859 (24597, 26634)	65999 (52290, 82492)	296.8 (278.7, 307)	234.1 (186.7, 290.9)	-21.1 (-37.4, -2.8)
Central Sub-Saharan Africa	73256 (61934, 84984)	149151 (118950, 183436)	410.9 (351.6, 468.3)	361.4 (292.1, 444.2)	-12.1 (-27.7, 7.2)
Republic of Angola	13695 (11313, 16496)	31833 (24654, 39289)	435.9 (367, 518.8)	364.1 (289.8, 447.6)	-16.5 (-35.5, 8.7)
Central African Republic	4777 (3945, 5668)	7621 (5825, 9710)	523.2 (441.7, 606.6)	456.8 (358.2, 556.6)	-12.7 (-28.8, 4.8)
Republic of the Congo	4643 (3933, 5452)	8368 (6703, 10206)	544.2 (468, 629.2)	413.5 (342.4, 487.6)	-24 (-36.8, -7.6)
Democratic Republic of the Congo	47260 (38298, 56786)	97266 (75161, 123949)	385.1 (315.1, 453.6)	352.5 (275.2, 445.5)	-8.5 (-29.3, 16.4)
Republic of Equatorial Guinea	805 (648, 977)	1283 (876, 1759)	501.3 (408, 599.8)	330 (238.7, 438.1)	-34.2 (-51.3, -8.7)
Gabonese Republic	2076 (1742, 2467)	2780 (2152, 3468)	419.4 (355.2, 498.7)	344.8 (275.5, 421.8)	-17.8 (-33.6, 3.8)
East Asia	2576813 (2280964, 2859920)	5231174 (4452990, 6056337)	403.3 (358.4, 445.9)	276.2 (235.1, 318.3)	-31.5 (-42.4, -18.1)
People's Republic of China	2497062 (2206621, 2780395)	5085458 (4311063, 5904298)	407.7 (361.4, 452.1)	280.1 (237.9, 323.9)	-31.3 (-42.4, -17.4)
Democratic People's Republic of Korea	48468 (37984, 58996)	106571 (87571, 126680)	383.4 (302.7, 455.9)	358.9 (296.6, 426.3)	-6.4 (-24, 17.4)
Taiwan (Province of China)	31283 (30098, 32091)	39146 (34278, 42155)	252.1 (236.9, 260.4)	89.4 (78.7, 95.8)	-64.5 (-67.2, -62.5)
Eastern Europe	1396707 (1336338, 1422675)	1490606 (1348329, 1620589)	570.1 (540.1, 583.5)	421.6 (381.3, 458.3)	-26 (-31.4, -20.5)
Republic of Belarus	60596 (57107, 62440)	76018 (64619, 87746)	496.6 (465.5, 512.1)	466.9 (396.6, 539.2)	-6 (-20.1, 8.7)
Republic of Estonia	11237 (10638, 11605)	7252 (6305, 8033)	579.5 (545.5, 598.7)	223.7 (195.9, 247.8)	-61.4 (-65.1, -57.5)
Republic of Latvia	19969 (18963, 20607)	14911 (13233, 16316)	577.1 (545, 596.5)	330.5 (294.1, 362)	-42.7 (-47.7, -38)
Republic of Lithuania	21557 (20416, 22228)	20227 (17988, 22140)	491.1 (464.3, 507.2)	302.7 (272.3, 331.5)	-38.4 (-43.9, -33.2)
Republic of Moldova	20851 (20075, 21394)	21918 (19953, 23889)	605.1 (575.8, 622)	363.5 (331.3, 395.8)	-39.9 (-44.6, -34.8)
Russian Federation	900491 (861889, 917006)	939256 (864841, 1010874)	579.5 (549.1, 593.1)	394.8 (363.6, 424.8)	-31.9 (-36.1, -27.6)
Ukraine	362006 (346443, 370151)	411023 (320290, 505529)	565.8 (535.2, 580.3)	521.5 (405.7, 642.5)	-7.8 (-27.3, 12.8)
Eastern Sub-Saharan Africa	210769 (194576, 228570)	356706 (321397, 398574)	330.3 (302.7, 356.9)	256 (231.4, 285.4)	-22.5 (-29.8, -13.4)
Republic of Burundi	9552 (7689, 11438)	11266 (9095, 13669)	450.6 (365.4, 535.3)	284.4 (230, 346.3)	-36.9 (-49.5, -18)
Union of the Comoros	577 (450, 711)	1018 (811, 1278)	349 (283.8, 422.8)	248 (199, 308)	-28.9 (-44.6, -6.7)
Republic of Djibouti	336 (259, 425)	1308 (993, 1703)	321.3 (256.8, 394.4)	278.9 (221.1, 352.3)	-13.2 (-34.4, 16)
State of Eritrea	3917 (3226, 4668)	7027 (5467, 8898)	435.5 (367.6, 509.9)	335.3 (272.5, 408.6)	-23 (-37.1, -6.3)
Federal Democratic Republic of Ethiopia	58002 (50698, 68834)	69072 (59589, 79851)	341.6 (299.3, 392.4)	184.5 (159.3, 213.3)	-46 (-56.2, -35.9)
Republic of Kenya	13304 (10820, 15471)	37904 (30758, 46409)	192.9 (155.6, 225.4)	218.9 (176.5, 268.5)	13.5 (-6.7, 40.6)
Republic of Madagascar	20860 (18837, 23010)	37628 (29042, 47282)	459.1 (414.1, 504.9)	415.7 (326.7, 510.4)	-9.4 (-29.3, 12.8)
Republic of Malawi	9594 (8429, 10804)	18374 (15649, 21696)	297.2 (260.5, 336.4)	301.1 (259.4, 351.3)	1.3 (-14.7, 21.3)
Republic of Mozambique	16408 (14379, 18837)	33677 (25907, 41702)	333.9 (294.8, 378.1)	370.6 (286.3, 452.7)	11 (-16.1, 36.7)
Republic of Rwanda	12314 (10590, 14251)	12385 (9732, 15752)	508.4 (442.6, 584)	254.6 (201, 321.1)	-49.9 (-60.3, -35.9)
Federal Republic of Somalia	7739 (6046, 9610)	14663 (10824, 19176)	386.8 (309.3, 466.2)	299.2 (222.2, 380.9)	-22.7 (-40.8, -2.1)
Republic of South Sudan	7469 (6061, 9289)	8701 (6515, 11420)	324 (264.3, 397.3)	270.6 (209, 341.2)	-16.5 (-35.6, 9.2)
Republic of Uganda	16259 (13219, 20014)	27121 (21730, 34223)	292.2 (237.1, 355.4)	222.8 (180.4, 277.7)	-23.8 (-41.3, -0.1)
United Republic of Tanzania	25991 (22620, 29503)	56908 (47315, 70470)	281.9 (246.9, 317.8)	267.7 (225.7, 326.9)	-5.1 (-21.9, 19.3)
Republic of Zambia	8294 (7118, 9555)	19343 (14693, 24367)	352.7 (304.4, 403.6)	354 (276, 430.5)	0.4 (-21.5, 25.1)
High-income Asia Pacific	358928 (325960, 375092)	455990 (363781, 507729)	204.1 (182.1, 214.9)	73.4 (61.3, 79.8)	-64 (-66.4, -62.5)
Brunei Darussalam	310 (283, 344)	496 (443, 556)	355 (322.8, 391.6)	198.5 (176.9, 222.7)	-44.1 (-50.8, -35.6)
Japan	283723 (254249, 297702)	371917 (292380, 416609)	186.4 (164.9, 196.8)	72.5 (60.8, 78.8)	-61.1 (-63.2, -59.8)
Republic of Korea	69566 (63580, 73806)	77286 (63726, 86294)	330.2 (297.6, 353.1)	85.9 (70.7, 95.9)	-74 (-76.9, -71.6)
Republic of Singapore	5328 (5105, 5467)	6291 (5606, 6712)	275 (259.5, 283.5)	75.8 (67.4, 80.9)	-72.4 (-74.4, -71)
High-income North America	943627 (849457, 988434)	981390 (850396, 1048167)	260.4 (234.8, 272.6)	139.5 (122.5, 148.1)	-46.4 (-48.2, -45.3)
Canada	74193 (68204, 77148)	75621 (65538, 81313)	232.8 (213.2, 242.5)	92.8 (81.5, 99.2)	-60.1 (-62.2, -58.7)
Greenland	112 (104, 120)	100 (88, 118)	445.6 (409.2, 480)	185.4 (161.1, 218.7)	-58.4 (-64.1, -51.6)
United States of America	869300 (780978, 911407)	905653 (785640, 966939)	263.2 (237.2, 275.8)	145.3 (127.7, 154.3)	-44.8 (-46.5, -43.7)
North Africa and the Middle East	750006 (698871, 796221)	1354626 (1210244, 1496618)	529.2 (486.4, 560.6)	363.5 (322.3, 397.3)	-31.3 (-37.1, -25)
Islamic Republic of Afghanistan	45396 (36453, 54432)	47557 (38096, 57568)	746.1 (609.1, 884.7)	567.4 (460.6, 672.2)	-23.9 (-38.1, -6)
People's Democratic Republic of Algeria	45402 (40240, 51069)	97643 (82937, 113387)	570 (509.2, 632.3)	392.6 (340.3, 448.7)	-31.1 (-40.8, -19.5)
Kingdom of Bahrain	718 (677, 761)	1396 (1208, 1586)	637.4 (596.4, 674.5)	289.9 (257.3, 325)	-54.5 (-60, -48.3)
Arab Republic of Egypt	164978 (158655, 172941)	275665 (234643, 323108)	798.8 (756.6, 839.5)	612.1 (530, 700.1)	-23.4 (-34.6, -11.9)
Islamic Republic of Iran	86527 (81551, 91001)	169582 (154574, 180461)	446.3 (411.5, 471.3)	254.5 (230.2, 271.3)	-43 (-45.9, -40.1)
Republic of Iraq	39014 (33523, 44756)	87555 (70317, 102687)	507.9 (436.1, 582.8)	477.6 (397.1, 553.4)	-6 (-25.8, 13.7)
Hashemite Kingdom of Jordan	4546 (3969, 5195)	11652 (9620, 14064)	419.6 (366.5, 479.2)	213.4 (177.5, 252.6)	-49.2 (-58.7, -36.5)
State of Kuwait	1543 (1424, 1646)	4201 (3483, 5031)	302.2 (273.5, 323.2)	167.8 (138, 200.2)	-44.5 (-53.3, -34.2)
Lebanese Republic	8140 (7058, 9654)	10855 (9133, 12611)	441.7 (381.2, 521.6)	164.5 (138.9, 191.8)	-62.8 (-69.3, -55.2)
State of Libya	5348 (4339, 6286)	14048 (11138, 17773)	292.1 (233.6, 345.4)	313.7 (251.1, 390.8)	7.4 (-15.7, 41)
Kingdom of Morocco	67431 (58948, 75052)	134920 (110214, 150427)	522.4 (453.8, 578.8)	463.2 (379.8, 513.3)	-11.3 (-28.4, 2.6)
Sultanate of Oman	3108 (2441, 3811)	4618 (3832, 5480)	524 (415.3, 637.6)	326.4 (272.3, 379.8)	-37.7 (-52.2, -17.6)
Palestine	4163 (3458, 5007)	6461 (5648, 7232)	568.2 (477.6, 676.4)	356.8 (311.7, 397.1)	-37.2 (-48.9, -24)
State of Qatar	399 (345, 461)	1048 (817, 1312)	610 (535.1, 685.8)	207 (166.7, 247.5)	-66.1 (-73, -58.2)
Kingdom of Saudi Arabia	23580 (18359, 29083)	54791 (45276, 65801)	471.8 (367.4, 576.7)	353.3 (305.3, 408.4)	-25.1 (-40.5, -0.3)
Republic of Sudan	53530 (44463, 62765)	74499 (59370, 97051)	629.9 (527.3, 733.4)	445.7 (363.7, 569.4)	-29.3 (-43.8, -9.4)
Syrian Arab Republic	28726 (24188, 34094)	51846 (40783, 65825)	602.1 (507.3, 708.3)	521.6 (422.6, 639.1)	-13.4 (-34.1, 14.3)
Republic of Tunisia	15785 (13906, 17637)	35009 (27179, 44423)	403.7 (352.5, 449.3)	300.7 (234.7, 378.2)	-25.5 (-42.3, -2.9)
Republic of Turkey	122591 (111143, 133494)	205740 (171192, 240575)	413.6 (370.4, 449.5)	247.9 (206.2, 288.5)	-40.1 (-49.9, -30.3)
United Arab Emirates	1609 (1307, 1978)	5123 (4113, 6192)	473.2 (391.5, 567.4)	314.1 (261.1, 366.1)	-33.6 (-42, -24.6)
Republic of Yemen	27064 (21809, 33255)	59153 (46524, 74392)	646.8 (526.5, 780.4)	517.8 (415.8, 637.9)	-19.9 (-37.3, 3.6)
Oceania	11290 (9435, 13443)	24587 (20668, 28675)	449.5 (384.7, 525.9)	377.8 (321.2, 438.3)	-15.9 (-29.1, -0.2)
Independent State of Samoa	344 (289, 395)	16622 (13257, 20730)	467.8 (394.2, 529.1)	336.1 (271.2, 411.3)	-8.6 (-24.6, 14.6)
American Samoa	69 (63, 75)	139 (122, 161)	369.6 (340.1, 395.9)	335.7 (295.9, 383.6)	-9.2 (-21, 4.1)
Cook Islands	45 (41, 50)	60 (50, 69)	419.8 (382.9, 459.5)	240.4 (202.4, 278.6)	-42.7 (-51.9, -31.8)
Republic of Fiji	1655 (1462, 1894)	2815 (2225, 3485)	521.3 (464.3, 590)	450.5 (364.4, 544.6)	-13.6 (-30.6, 8.1)
Guam	208 (196, 220)	395 (350, 435)	375.7 (347.6, 395.2)	186.1 (166.2, 204.4)	-50.5 (-54.9, -45.5)
Republic of Kiribati	152 (127, 177)	275 (225, 341)	447.6 (366.6, 519.6)	434.4 (370.1, 515)	-2.9 (-20.2, 23.5)
Republic of the Marshall Islands	87 (78, 97)	159 (125, 198)	601.8 (540.6, 667.2)	546.8 (446.7, 659.4)	-9.1 (-25.5, 9.7)
Federated States of Micronesia	276 (229, 323)	331 (266, 414)	624.6 (521.8, 728.3)	532 (440.4, 646.2)	-14.8 (-32.1, 8)
Republic of Nauru	32 (26, 38)	39 (31, 48)	780 (651.5, 901.4)	748.3 (624.7, 892.4)	-4.1 (-20.8, 19.8)
Republic of Niue	11 (10, 13)	9 (7, 10)	483.9 (412.3, 565.6)	430 (367.5, 489.9)	-11.1 (-26.6, 6.8)
Northern Mariana Islands	46 (38, 57)	118 (103, 124)	324.7 (278.3, 389.2)	286.9 (255.5, 306.4)	-11.6 (-27.2, 3.7)
Republic of Palau	41 (34, 49)	72 (59, 86)	476 (401.7, 561.7)	407.2 (341.7, 476.1)	-14.4 (-31.4, 6.4)
Independent State of Papua New Guinea	6439 (5006, 8192)	15878 (12501, 19674)	413.4 (328.7, 516)	364.9 (289, 449.1)	-11.7 (-33.9, 19.1)
Solomon Islands	670 (471, 846)	1591 (1292, 2015)	610.2 (490.4, 727)	550.3 (464.4, 674.5)	-9.8 (-28.1, 13.8)
Tokelau	6 (5, 7)	6 (5, 7)	509.4 (433.9, 591)	384.7 (312.8, 461.2)	-24.5 (-38.2, -7.1)
Kingdom of Tonga	126 (107, 144)	188 (154, 226)	260.5 (219.9, 298.5)	243.3 (200, 291.9)	-6.6 (-27.5, 19.6)
Tuvalu	36 (31, 41)	46 (40, 54)	637.8 (553.8, 716.7)	502.8 (440.1, 581.6)	-21.2 (-31.4, -9.6)
Republic of Vanuatu	324 (270, 401)	813 (690, 948)	606.5 (517.8, 718.4)	545 (465.7, 625)	-10.1 (-25.9, 8.6)
South Asia	1554655 (1402490, 1674222)	3666331 (3381322, 3957645)	302.5 (271.1, 326.8)	278.1 (256.5, 299.6)	-8 (-17.7, 2.6)
People's Republic of Bangladesh	176069 (156197, 196602)	377200 (309884, 453235)	396.7 (351.6, 442.7)	313 (259.9, 372.7)	-21.1 (-36.8, -2.5)
Kingdom of Bhutan	597 (468, 743)	1302 (1065, 1530)	282.1 (216.4, 353)	233.1 (192.1, 272.4)	-17.4 (-36.3, 9.4)
Republic of India	1191436 (1057016, 1303879)	2873266 (2625504, 3154720)	287.5 (252.1, 315.6)	267.6 (243.2, 293.4)	-6.9 (-18.4, 5.4)
Federal Democratic Republic of Nepal	26752 (22424, 32156)	55697 (46416, 67871)	325.6 (271, 389.5)	278.3 (234.6, 336.6)	-14.5 (-32.9, 9.9)
Islamic Republic of Pakistan	159801 (133369, 177964)	358866 (304004, 436445)	310.9 (258, 348.5)	342 (292.3, 416.6)	10 (-8.2, 35.8)
Southeast Asia	779349 (707641, 847500)	1721549 (1556629, 1858463)	355.5 (318.9, 388.7)	302.9 (273.2, 326.1)	-14.8 (-23.9, -3.4)
Kingdom of Cambodia	15703 (13631, 18284)	33261 (27055, 39723)	411.5 (357.5, 479.1)	341.8 (281, 398.6)	-16.9 (-34.2, 1.8)
Republic of Indonesia	300490 (256841, 336013)	765660 (647217, 876429)	356 (295.9, 409.4)	409.9 (343.7, 459.4)	15.1 (-6.4, 39.6)
Lao People's Democratic Republic	11117 (9113, 13252)	15701 (12642, 19048)	610.9 (502.4, 717.9)	410.9 (337.2, 485.9)	-32.7 (-47.5, -13.1)
Malaysia	29630 (28066, 30853)	66779 (62583, 70162)	347.1 (325.9, 363.5)	267.9 (250.1, 282.6)	-22.8 (-27.1, -17.7)
Republic of Maldives	322 (298, 346)	513 (426, 599)	434 (394.6, 466.6)	180.5 (150.5, 208.3)	-58.4 (-64.8, -51.5)
Republic of Mauritius	2960 (2842, 3054)	3554 (3292, 3692)	469.8 (447.8, 486)	210.5 (194.9, 219.2)	-55.2 (-57.6, -53.6)
Republic of the Union of Myanmar	111990 (91339, 134638)	152058 (126346, 184972)	551 (456.3, 654.9)	363.6 (306.7, 439)	-34 (-49, -14.3)
Republic of the Philippines	83879 (77590, 89946)	225939 (193234, 259965)	363.2 (337.5, 388.7)	315.1 (272, 359.8)	-13.2 (-24.9, 0.2)
Republic of Seychelles	210 (196, 222)	242 (220, 265)	374.3 (349.1, 396)	238.7 (217.3, 260.5)	-36.2 (-41.6, -29.5)
Democratic Socialist Republic of Sri Lanka	31473 (29226, 33607)	54797 (38914, 71914)	370.6 (342.9, 394.5)	232.3 (167.8, 299.8)	-37.3 (-55.4, -18.6)
Kingdom of Thailand	66429 (58187, 74763)	136761 (108137, 167205)	225.5 (196.6, 254.1)	128 (101.4, 156.2)	-43.2 (-55.4, -28)
Democratic Republic of Timor-Leste	846 (678, 1010)	2782 (2235, 3395)	368.5 (296.2, 433.2)	380.4 (308.9, 458.4)	3.2 (-20.6, 34.9)
Socialist Republic of Viet Nam	123174 (101742, 151159)	261099 (218650, 297128)	342.2 (283, 418.5)	310.5 (262.2, 351.1)	-9.3 (-28.4, 13)
Southern Latin America	133794 (127096, 137287)	124512 (113757, 131073)	315.6 (297.1, 324.9)	137.6 (126.1, 144.6)	-56.4 (-58, -54.9)
Eastern Republic of Uruguay	12030 (11340, 12435)	9942 (8921, 10562)	314.2 (295.3, 325)	154.8 (140.9, 163.7)	-50.7 (-52.9, -48.8)
Argentine Republic	99491 (94663, 102344)	87402 (80136, 91960)	335.8 (316.4, 346.2)	151.1 (138.8, 158.9)	-55 (-56.8, -53.3)
Republic of Chile	22267 (21231, 22885)	27161 (24492, 28692)	251.3 (237.5, 259.3)	103.8 (93.8, 109.5)	-58.7 (-60.9, -57.2)
Southern Sub-Saharan Africa	58586 (52618, 63696)	130763 (121926, 139194)	245.7 (217.5, 269.4)	275.1 (256.6, 292.1)	12 (4.1, 25.2)
Republic of Botswana	1668 (1288, 2068)	2890 (2350, 3532)	387.5 (304, 473.9)	256.6 (215.5, 309)	-33.8 (-48.3, -10)
Kingdom of Eswatini	864 (721, 1018)	1612 (1187, 2146)	376.7 (314.3, 445.6)	360.7 (274.8, 460.5)	-4.2 (-28.5, 27)
Kingdom of Lesotho	2083 (1685, 2500)	3653 (2805, 4566)	287.5 (230.1, 348.4)	412.9 (327.8, 500.9)	43.6 (7.7, 96.3)
Republic of Namibia	1976 (1708, 2247)	3752 (2964, 4602)	392.9 (340.2, 447.1)	347.4 (281.8, 418.2)	-11.6 (-29.5, 9.6)
Republic of South Africa	43378 (38357, 47166)	98605 (92046, 104928)	230.5 (201.4, 252.2)	256.1 (236.8, 272.3)	11.1 (3, 24.3)
Republic of Zimbabwe	8617 (7189, 9989)	20252 (16536, 24375)	277.2 (229.4, 320.7)	370.1 (309.7, 430.8)	33.5 (7.5, 68.7)
Tropical Latin America	263566 (250152, 270019)	385370 (351507, 405260)	331.7 (308, 343)	154.6 (140.5, 162.8)	-53.4 (-55.3, -52.1)
Federative Republic of Brazil	258237 (245353, 264534)	374765 (342776, 394079)	333.7 (310.1, 345)	153.7 (140.1, 161.9)	-53.9 (-55.7, -52.5)
Republic of Paraguay	5329 (4713, 5948)	10606 (8239, 13103)	258.3 (226.9, 288.5)	194.6 (151.5, 240.1)	-24.7 (-40.8, -7.9)
Western Europe	1665937 (1523644, 1730875)	1262954 (1058669, 1367244)	280.7 (256.2, 292.2)	106.8 (91.8, 114.4)	-62 (-64.3, -60.7)
Principality of Andorra	79 (59, 104)	156 (115, 200)	167.5 (126.4, 215.5)	88.3 (65.6, 112.9)	-47.3 (-62.4, -25.8)
Republic of Austria	40057 (36825, 41552)	29290 (24708, 31703)	326.9 (299.2, 339.5)	128.6 (110.6, 138.2)	-60.7 (-63.3, -59)
Kingdom of Belgium	39795 (36237, 41720)	26402 (21716, 28854)	254.3 (230.8, 266.9)	90 (76.3, 97.4)	-64.6 (-66.8, -63)
Republic of Cyprus	2803 (2619, 2978)	3038 (2625, 3395)	554.7 (518.8, 587.5)	184.9 (162.5, 205.1)	-66.7 (-70.2, -63)
Kingdom of Denmark	27160 (25237, 28152)	12978 (11142, 13937)	314.1 (292.6, 325.5)	95.2 (82.5, 101.9)	-69.7 (-71.6, -68.3)
Republic of Finland	24339 (22436, 25277)	22551 (18811, 24561)	338.8 (311, 352.7)	143.3 (122, 154.8)	-57.7 (-61, -55.6)
French Republic	176601 (160734, 184160)	152728 (128083, 166336)	199.4 (181.5, 208)	78.7 (67.7, 85)	-60.6 (-63.2, -58.7)
Federal Republic of Germany	440017 (402261, 458425)	319026 (268347, 345701)	332.1 (303, 346.6)	132.8 (114, 142.7)	-60 (-62.5, -58.5)
Hellenic Republic	46916 (43321, 48610)	48088 (41397, 51375)	328.5 (301.4, 341.5)	154 (136.3, 163)	-53.1 (-54.9, -51.7)
Republic of Iceland	755 (682, 801)	701 (572, 780)	247.9 (225, 262.7)	101.4 (84.3, 112.3)	-59.1 (-62.8, -55.5)
Ireland	13986 (13182, 14444)	8293 (6971, 9021)	356.1 (331.6, 368.8)	97.6 (82.5, 105.9)	-72.6 (-75, -70.9)
State of Israel	12208 (11305, 12659)	10554 (8916, 11439)	270.3 (247.8, 281.8)	75.2 (64.4, 81.2)	-72.2 (-74.3, -70.6)
Republic of Italy	222138 (199096, 233114)	216272 (175532, 238313)	257.3 (229.2, 271.1)	109.1 (91, 118.9)	-57.6 (-60.3, -56.1)
Grand Duchy of Luxembourg	1718 (1623, 1783)	1275 (1107, 1404)	326.2 (306.5, 338.6)	103.9 (91.1, 114.5)	-68.1 (-70.9, -65.3)
Republic of Malta	1319 (1230, 1385)	1307 (1102, 1452)	333.7 (307.7, 351.2)	117.5 (100.7, 130)	-64.8 (-67.9, -61.7)
Principality of Monaco	200 (157, 233)	150 (119, 182)	247.2 (195.9, 287.4)	123.1 (98.2, 149.2)	-50.2 (-60.2, -35.3)
Kingdom of the Netherlands	51266 (46643, 53650)	40439 (34331, 43711)	251.8 (228.8, 263.8)	100.9 (86.4, 108.8)	-59.9 (-62.3, -58.3)
Kingdom of Norway	21254 (19409, 22228)	10906 (9160, 11813)	283.7 (260.5, 296.2)	90 (76.9, 96.9)	-68.3 (-70.5, -67)
Portuguese Republic	46500 (43626, 48030)	32680 (27559, 35442)	375.4 (348.8, 389.1)	106.4 (92.1, 114.3)	-71.7 (-73.5, -70.4)
Republic of San Marino	72 (62, 81)	70 (49, 94)	193.2 (164.5, 217.8)	66.9 (45.4, 90.4)	-65.4 (-75.2, -53.2)
Kingdom of Spain	124681 (113975, 130453)	115285 (94382, 126132)	236.7 (215.3, 248.2)	90 (75.9, 97.3)	-62 (-64.2, -60.2)
Kingdom of Sweden	46749 (42812, 48805)	29625 (24644, 33050)	279.5 (256.8, 291.6)	107.4 (91.3, 119.4)	-61.6 (-65.4, -58.4)
Swiss Confederation	26458 (23954, 27739)	20297 (16421, 22375)	234.3 (213.3, 245.3)	84.1 (69.6, 91.8)	-64.1 (-67.4, -62.1)
United Kingdom of Great Britain and Northern Ireland	297499 (277475, 306303)	159730 (139629, 169392)	315.8 (294.6, 325.4)	106.6 (94.6, 112.4)	-66.2 (-67.9, -65.4)
Western Sub-Saharan Africa	267218 (238738, 296709)	466045 (403943, 528620)	354.3 (315.9, 393.3)	291.9 (260.9, 326.2)	-17.6 (-27.7, -5.5)
Republic of Benin	5982 (5293, 6780)	11863 (10058, 14035)	327.2 (288.4, 372.6)	277.4 (238.4, 323.2)	-15.2 (-28.2, 2.8)
Burkina Faso	10643 (9232, 12326)	22379 (18746, 26771)	288.1 (250.4, 331.8)	288.1 (244.3, 343.1)	0 (-17.2, 24.2)
Republic of Cabo Verde	540 (467, 612)	1139 (961, 1318)	225.3 (194.6, 256.2)	274 (231.1, 315.8)	21.6 (-1.4, 52.1)
Republic of Cameroon	12112 (10307, 14172)	32549 (25085, 42444)	324.3 (279.7, 375.8)	318.2 (256.7, 408.7)	-1.9 (-21.1, 29.7)
Republic of Chad	8219 (7031, 9378)	16593 (13047, 20576)	316.1 (270.5, 362.6)	334.5 (270.6, 402.2)	5.8 (-14.6, 31.9)
Republic of Côte d'Ivoire	11435 (9510, 13597)	29124 (22858, 37210)	357 (307.1, 409.6)	326.4 (271.2, 405.2)	-8 (-21.3, 11.4)
Republic of the Gambia	1050 (840, 1300)	3230 (2532, 3896)	353.3 (289, 431.9)	386.8 (306.4, 464.1)	9.5 (-17, 39.9)
Republic of Ghana	22994 (19309, 27275)	48155 (38389, 58063)	437.3 (372.8, 513.3)	355.3 (286.8, 425.3)	-18.8 (-36.6, 3)
Republic of Guinea-Bissau	1817 (1492, 2193)	2730 (2153, 3250)	516 (429.9, 609.4)	469.9 (379.5, 543.7)	-8.9 (-29.1, 13.2)
Republic of Guinea	10078 (8410, 11839)	550 (468, 654)	330.7 (275.1, 390.6)	430.4 (371.5, 505.3)	1.6 (-20.6, 34.3)
Republic of Liberia	3700 (3167, 4312)	5792 (4629, 7469)	356.9 (308.2, 410.3)	334.4 (275.2, 419.5)	-6.3 (-24.8, 22.1)
Republic of Mali	9841 (8672, 11176)	17755 (14455, 21412)	292 (254.5, 330)	239.9 (200.4, 285.6)	-17.8 (-32.9, 0.2)
Islamic Republic of Mauritania	3727 (3102, 4461)	5698 (4526, 7122)	424.4 (352.3, 504.9)	311.4 (248.9, 386.9)	-26.6 (-41.4, -6.7)
Republic of the Niger	7015 (5741, 8352)	17576 (13464, 22193)	295.4 (242.2, 348.9)	265.5 (210.6, 329)	-10.1 (-26.8, 12.3)
Federal Republic of Nigeria	136164 (114977, 160014)	190897 (157963, 230762)	359.9 (304.9, 420.6)	262.5 (226.8, 308)	-27.1 (-40.2, -8.5)
Democratic Republic of São Tomé and Príncipe	166 (148, 184)	272 (236, 315)	282 (253.1, 310.5)	296.9 (263.6, 340)	5.3 (-7.6, 20.4)
Republic of Senegal	10731 (9145, 12385)	21857 (17613, 26979)	373.8 (319.9, 426.8)	336 (274.9, 409.7)	-10.1 (-28.6, 11.6)
Republic of Sierra Leone	7145 (5990, 8419)	11281 (8954, 14096)	373.9 (314.2, 435)	344.8 (280.3, 418.7)	-7.8 (-25, 16.5)
Togolese Republic	3849 (3336, 4469)	10529 (8049, 13411)	365.3 (318.7, 423.2)	353 (281.6, 438.5)	-3.4 (-23.5, 23.2)

From 1990 to 2021, the ASIR of CVD showed a decreasing trend globally, except in Central Asia and East Asia. The region with the largest decrease in ASIR was high-income North America, where the rate declined from 986.6 per 100,000 in 1990 to 670.2 per 100,000 in 2021, a reduction of 32.1% (Table 1). Similarly, from 1990 to 2021, the ASDR of CVD also showed a decreasing trend globally, except in Southern Sub-Saharan Africa. The region with the largest decrease in ASDR was Australasia, where the rate declined from 280 cases per 100,000 in 1990 to 94.2 cases per 100,000 in 2021, a reduction of 66.4% (Table 1) (Table 1 provides detailed data for the 21 GBD regions, Table 2 provides detailed data for the 204 countries, and Figure 1 provides a global view of the CVD ASIR and ASDR).

In summary, we find that Asia is the hardest hit by CVD due to high population density and the progression of population aging. The regions with the highest number of CVD incidence and deaths are East and South Asia, especially China and India, which are the most populous countries. Secondly, diets high in salt, fat, and sugar are prevalent in East and South Asia, and these are risk factors for CVD [10]. In addition, these regions and countries suffer from unbalanced economic development and uneven distribution of healthcare resources, with a large number of patients with CVD in the population that are not adequately reported and treated. In 2021, we find that regions with higher ASIR for CVD are dominated by low- and middle-income developing countries. Similarly, these regions also have higher ASDR for CVD. The most likely explanation is that the region has experienced rapid economic development and urbanization in recent decades, resulting in lifestyle changes such as westernization of dietary habits, reduced physical activity, smoking, and increased body mass index, all of which contribute to a higher ASIR for CVD [11]. However, these places also suffer from financial inequality and unequal distribution of healthcare resources, making it difficult for low-income residents to get high-quality healthcare services and health education [12,13]. The presence of a large number of patients with CVD in the population who do not receive standardized care makes the ASDR high. In contrast, the ASIR and ASDR for CVD in high-income regions, represented by the high-income Asia-Pacific region, Australasia, high-income North America, and Western Europe, are both at lower levels. The high-income Asia Pacific area has the lowest ASIR and ASDR for CVD, high-income North America has the highest reduction in ASIR, and Australasia has the largest decrease in ASDR. The possible reasons for this are as follows. Firstly, these locations are primarily developed countries, which often have tighter environmental protection regulations, resulting in healthier living and working conditions. Secondly, the residents of these nations are typically highly informed about CVD risk factors and preventative techniques, allowing them to comprehend and adopt healthy lifestyles more effectively [14]. These help to reduce ASIR in CVD. Furthermore, people from high-income nations have higher levels of income and easier access to healthcare [8]. Finally, high-income nations have improved and sophisticated healthcare systems that ensure enough access to high-quality medical services, such as early detection, prompt treatment, and effective rehabilitation approaches [8,11]. These help reduce the ASDR of CVD. It is worth noting that, globally, only Southern Sub-Saharan Africa has shown an increasing trend in CVD deaths over the past 30 years. CVD is the second biggest killer in sub-Saharan Africa, the first being poverty-related malnutrition and infectious diseases [4]. However, these nations frequently lack national policies and continue to have inadequate, underfunded, and under-resourced primary healthcare systems, resulting in a higher ASDR for CVD [15].

Association with SDI

Figure 2 shows ASIR and ASDR for CVD across time between the global and five SDI regions from 1990 to 2021. Overall, the ASIR of CVD has shown a declining trend globally, as well as in high SDI and high-middle SDI regions. In contrast, the ASIR in middle SDI, low-middle SDI, and low SDI regions has remained relatively stable. The ASIR in high SDI regions is significantly lower than the global average and has shown the largest decline. In high-middle SDI regions, the ASIR did not change much before 2000 but has since shown a clear downward trend (Figure 2). During the same period, the ASDR for CVD has shown a declining trend globally and in all five SDI regions. The ASDR in high SDI regions is significantly lower than the global average and has shown the largest decline. The differences in ASDR among the other four SDI regions are relatively small (Figure 2).

We observed that among the five SDI regions, the ASIR and ASDR for CVD were the lowest and decreased the greatest in the high SDI regions. For example, in Sweden, Australia, Japan, and Canada, the common characteristics of these countries are a well-developed healthcare system, excellent health education, and a high rate of promotion of public health policies. It is noteworthy that in high-middle SDI regions, the ASIR for CVD remained stable until 2000, while there was a significant downward trend after 2000, which may be related to the promotion and implementation of public health policies in these places. For example, after 2000, the World Health Organization launched a global campaign against smoking. With the publication of the Framework Convention on Tobacco Control (FCTC) on May 21, 2003, as of January 2005, nearly 90% of the world's countries have signed the FCTC [16]. In 2006, the Russian government introduced a series of alcohol control measures. Furthermore, the public health departments of Poland, Russia, Chile, and Malaysia have implemented health programs, expanded health insurance coverage, and increased funding in primary healthcare facilities. These measures are effective in reducing the risk of CVD by providing health guidance and support. In contrast, middle SDI regions, low-middle SDI regions, and low SDI regions with low SDI face problems such as insufficient investment in medical resources, weak health awareness, and low accessibility to medical care, which make the morbidity and mortality of CVD remain high [8].

## Discussion

In this study, we systematically assessed global trends and regional differences in CVD incidence and mortality from 1990 to 2021. We also evaluated disease trends across five SDI regions worldwide. First, our findings indicate that from 1990 to 2021, the ASIR and ASDR for CVD decreased by 10.4% and 34.3%, respectively. However, the absolute number of CVD incident cases and deaths increased by 92.3% and 57.5%, reaching 66.81 million and 19.42 million cases, respectively. Additionally, we observed significant geographical and country-specific variations in CVD incidence and mortality. Developing countries in Central Asia, North Africa, and the Middle East also exhibit high incidence and death rates. Finally, the high SDI region has much lower ASIR and ASDR compared to the global average and has shown the most significant declines among the five SDI regions.

Studies based on GBD 2017 [2] and GBD 2019 [4] have reported differences in CVD across regions and countries worldwide. The GBD 2017 shows that the death rate from CVD is on a downward trend in most countries around the world. The decrease in CVD mortality between 1990 and 2017 was positively connected with the SDI, with higher SDI resulting in bigger declines in CVD mortality [2]. The GBD 2019 research found similar results, with ASIR and ASDR for CVD in high SDI regions falling the most dramatically between 1990 and 2019 [4]. Overall, the findings of this study are highly consistent with previous studies.

A key finding of this study is that there are significant geo-socioeconomic differences in CVD. This indicates that socioeconomic factors play an important role in the onset, progression, and regression of CVD. Geographic inequalities in socioeconomic status are linked to differences in the allocation of resources for healthcare and education, which leads to differences in disease perceptions, access to healthcare, living and working environments, and policymaker decisions [3,14,17,18]. In high-income countries, patients with CVD are more likely to be reported and treated due to their strong healthcare systems, adequate healthcare resources, and increased awareness of the disease [19]. In contrast, in low-income countries with limited access to healthcare, a significant proportion of patients with asymptomatic or insidious CVD may remain neglected [20]. A prospective study of over 150,000 participants from developing countries found that patients in low-income countries had the lowest cardiovascular risk but the highest rates of CVD, morbidity, and mortality, whereas patients in high-income countries had the opposite [21]. Therefore, recognizing the disease patterns of CVD prevalence among different countries and regions is essential for public health authorities to develop effective strategies to reduce the disease burden. The underlying principle of the theoretical framework for epidemiological transition is that the health status and spectrum of diseases that characterize different countries are related to their socioeconomic environment [22]. Nations at various phases of economic growth may have disease profiles with varying transitional stages in CVD spread [22]. Therefore, recognizing the features of the various transitory phases of the CVD epidemic in different nations is critical for guiding the identification of priority concerns in public health, resource allocation, and research in those countries. It is noteworthy that both previous studies [2] and the present study found that Uzbekistan has a high incidence and death rate of CVD. This might be attributed to Uzbekistan's poor medical resources, high smoking rates, a salt-, fat-, and sugar-rich diet, and a lack of social support and health education [23].

There are also limitations in this study. First, some of the heterogeneity in the reporting of observed and expected causes of early deaths in different regions may be artificial due to differences in data quality and the use of different methodologies and have changed over time. This change over time is not accurately captured in the creation of cause-of-death databases, which may be underestimating the burden of disease. Second, CVD includes a variety of subtypes, such as atrial fibrillation/flutter, coronary artery disease, and stroke, but this study did not analyze for subtypes, as well as lacked data related to treatment. For example, gender differences in CVD have been demonstrated, with men having a higher burden of ischemic heart disease and stroke than women and women having higher mortality rates from rheumatic heart disease and atrial fibrillation and flutter. Therefore, studies targeting subtypes of CVD are necessary.

## Conclusions

The ASIR and ASDR for CVD have been declining globally over the past 30 years as a result of health promotion in public health policies and technological advances in disease diagnosis and treatment. At the same time, the absolute number of CVD incidences and deaths worldwide continues to rise as a result of changes in lifestyle and risk factors due to globalization, as well as progress in population aging. The distribution of the burden of CVD varies considerably between regions and countries, which is related to socioeconomics, living and working environments, prevalence of risk factors, and accessibility of healthcare. The ASIR and ASDR for CVD in high SDI regions are much lower than in other SDI regions and have declined the most. This is linked to these countries' well-developed healthcare systems, strong health education, healthy lifestyles, and promotion of health programs. The accuracy of GBD estimates depends largely on the quality and quantity of data, and the relatively low coverage of high-quality CVD registries in countries with low SDI, as well as in low- and middle-income developing countries, renders the accuracy and comparability of data in these countries relatively low. It is suggested that these nations learn from successful strategies in developed nations while devising more effective ways to lessen the burden of CVD, taking into consideration their specific disease patterns and socioeconomic status. Finally, we recommend that there be further research on CVD subtypes and risk factors, which will help to develop more targeted public health strategies to promote global health.
